# Global Genomic Analysis of *Brucella* spp. Reveals Antimicrobial Resistance, Virulence, and Mobile Genetic Element Profiles from a One Health Perspective

**DOI:** 10.3390/microorganisms14102132

**Published:** 2026-09-22

**Authors:** Chenyang Han, Tao He, Yafei Liang, Bo Yang, Wenjuan Gong, Haihong Hao, Jinliang Sheng, Yu Pan

**Affiliations:** 1 Xinjiang Production and Construction Corps Key Laboratory for Efficient Utilization of Non-Grain Feed Resources, International Joint Research Center for Healthy Animal Farming, College of Animal Science and Technology, Shihezi University, Shihezi 832003, China; 18238863132@163.com (C.H.); ht960704@163.com (T.H.); 13899390597@163.com (Y.L.); 18089337984@163.com (B.Y.); 19399877826@163.com (W.G.); 2 National Reference Laboratory of Veterinary Drug Residues, Huazhong Agricultural University, Wuhan 430070, China; haohaihong@mail.hzau.edu.cn

**Keywords:** *Brucella* spp., antimicrobial resistance, virulence factors, global genomic surveillance

## Abstract

Brucellosis is a globally distributed zoonotic disease that poses a severe threat to human and animal health. However, the population structure, evolutionary dynamics, and antimicrobial resistance (AMR)-associated genomic characteristics of *Brucella* spp. across hosts, regions, and time periods remain insufficiently characterized. A total of 1760 publicly available *Brucella* spp. genomes were retrieved. After metadata screening, 701 genomes lacking required metadata were excluded, and 1059 genomes were retained for downstream analyses. Analyses included annotation of antimicrobial resistance genes (ARGs), virulence factors (VFs), and mobile genetic elements (MGEs), together with multilocus sequence typing (MLST), pan-genome analysis, and phylogenomic reconstruction. MLST assigned 836 genomes to 49 sequence types, whereas 223 remained unclassified. Environmental isolates showed relatively higher ARG annotation abundance in the analyzed dataset. Peptide resistance-associated annotations predominated (91.6%), largely due to *Bsui_mprF* and *bacA*, followed by β-lactam (4.3%) and aminoglycoside (2.6%), reflecting resistance-associated genomic annotations rather than phenotypically confirmed AMR. Several ARGs occurred in MGE-associated genomic contexts, suggesting potential mobility. VF profiles were broadly conserved overall, while selected determinants varied among species, geographic regions, and sources. Phylogenomic analysis revealed close relationships among isolates from different hosts, with ST8 and ST11 representing predominant lineages. These findings support integrated genomic surveillance across human, animal, and environmental sources.

## 1. Introduction

Brucellosis is a prevalent zoonotic disease caused by *Brucella* spp. and widely distributed worldwide [1]. In developing countries with relatively weak public health and veterinary disease-control systems, brucellosis remains endemic or causes recurrent outbreaks. Particularly in the Mediterranean region, the Middle East, Central Asia, and parts of Africa, a relatively high incidence has been reported [2,3,4]. Most human brucellosis cases are acquired through direct or indirect contact with infected animals and contaminated animal-derived products, or inhalation of infectious aerosols, highlighting the predominantly zoonotic transmission route of this disease [5]. In recent years, increased livestock movement and international trade, consumption of unpasteurized dairy products, and gaps in surveillance and veterinary control systems have contributed to the persistence or re-emergence of brucellosis in several regions [6,7,8]. In China, the reported incidence of brucellosis showed an overall upward trend from 2004 to 2021, accompanied by an expansion of affected areas from traditionally endemic northern regions to southern and inland regions [9,10]. Evidence has shown that the increase in human brucellosis cases may lag behind outbreaks of animal brucellosis, suggesting a close epidemiological association between brucellosis in livestock and humans [11]. Therefore, the persistent global burden of brucellosis has made it a major One Health challenge, causing substantial economic losses to livestock production and public health systems [12].

Against this One Health background, effective brucellosis control primarily depends on the management of animal reservoirs, while the diagnosis and treatment of human infections remain important components. For human infections, prolonged combination antibiotic regimens, particularly doxycycline plus rifampicin or doxycycline plus streptomycin/gentamicin, are generally recommended [13,14]. However, clinical treatment remains challenging because *Brucella* spp. are facultative intracellular pathogens capable of causing persistent and recurrent infections [15]. Disease may recur because *Brucella* spp. can persist intracellularly or remain in infected tissues [16,17]. Moreover, the development of antimicrobial resistance (AMR) in *Brucella* spp. requires attention, as previous studies have suggested that antibiotic use in clinical and animal-production settings may increase selection pressure for resistant bacteria and promote the persistence or dissemination of resistance determinants [18]. In this context, mobile genetic elements (MGEs) and horizontal gene transfer (HGT) have been recognized as potential mechanisms that may contribute to dissemination of antibiotic resistance genes (ARGs) across different hosts and environments [19].

With the decrease in sequencing costs and the expansion of genomic databases, genome-based analysis has been increasingly used to study the population structure and genetic characteristics of *Brucella* spp. [20]. Previous studies have mainly focused on specific species, such as *B. melitensis* and *B. abortus*, or on strains from particular regions or hosts [21,22,23]. Although classical *Brucella* species are highly similar at the genomic level, different species and lineages may still differ in host preference, virulence, and zoonotic potential [24,25,26]. However, comprehensive comparisons integrating isolates from human, livestock, wildlife, and environmental sources within a common genomic framework remain limited. Environmental and wildlife isolates are particularly underrepresented in previous comparative studies. In addition, the combined variation in population structure, antimicrobial resistance determinants, virulence factors, mobile genetic element-associated genes, and pan-genome characteristics across host sources, geographic regions, and sampling periods has not been fully characterized.

To address these gaps, we analyzed a global dataset comprising 1059 publicly available *Brucella* spp. genomes to characterize population structure and genomic variation across geographic regions, host origins, and dominant sequence types (STs). We further characterized the AMR profiles of *Brucella* spp. across ecological scales, including continents, hosts, and time periods, to explore the distribution of ARGs and their potential dissemination among humans, livestock, wildlife, and environmental sources. By integrating phylogenetic, pan-genome, VF and MGE analyses, this study provides a comprehensive genomic view of *Brucella* spp. from a One Health perspective. Particular attention was given to their evolutionary relationships, host-associated genomic features, and AMR dissemination potential.

## 2. Materials and Methods

### 2.1. Data Collection

This study aimed to create a global insight into the AMR in *Brucella* spp. across humans, livestock, wild animals, and environments, and reveal the genetic diversity and the evolution history. The spatiotemporal distribution of publicly available *Brucella* genomes was used to describe the geographic representation and clustering patterns. To ensure the comprehensiveness of the analyzed dataset, all genomes utilized in this study were obtained from the National Center for Biotechnology Information (NCBI, https://www.ncbi.nlm.nih.gov/, accessed on 21 September 2026) and Integrated Microbial Genomes (IMG, https://img.jgi.doe.gov/, accessed on 21 September 2026), with data download cutoff in July 2025. Genome records from the two databases were cross-checked using NCBI Assembly Accession numbers. Records sharing the same assembly accession were considered duplicates, and only one record was retained. After duplicate removal, 1760 non-redundant *Brucella* spp. genomes were retained for further screening.

*Brucella* genomes with complete metadata including isolation source, species, sampling date, host and geographical origin were screened for subsequent analyses. Genomes lacking any of these required metadata fields were excluded. A total of 701 genomes were excluded because of incomplete metadata (Table S11), leaving 1059 genomes for downstream analyses (Table S1). No additional genome-level quality filters based on assembly quality, genome completeness, contamination, genome length, or number of contigs were applied during selection of the primary 1059-genome dataset. Therefore, metadata completeness was not considered equivalent to genome quality. To assess the potential influence of genome quality on the principal analyses, an additional sensitivity analysis was performed using a high-quality (HQ) subset consisting of genomes with completeness ≥95% and contamination ≤5%. A total of 800 genomes met these criteria. ARG, VF, MGE-associated gene, pan-genome, and phylogenetic analyses were repeated using this HQ subset. Detailed procedures and results of the sensitivity analysis are provided in the Supplementary Methods, Supplementary Figures S12–S15, and Table S12.

### 2.2. Identification of ARGs, Virulence Factors and MLST Profiles

ARGs and VFs were identified by sequence similarity searches against the SARG database (v3.2.1) [27], the CARD database (v4.0.0) [28], and the VFDB database (2025) [29]. CARD was used as the primary ARG reference database, whereas SARG was used as a supplementary source. During database integration, SARG entries were cross-referenced against CARD using ARO identifiers. When a SARG entry corresponded to an ARO already represented in CARD, the CARD entry was retained and the overlapping SARG entry was not included again. SARG entries not represented in CARD according to the ARO-based comparison were retained as supplementary reference entries. The resulting nonredundant combined database was then used for ARG annotation. Protein sequences were queried against the ARG reference databases using BLASTP (v2.5.0), whereas nucleotide sequences were queried against the VFDB Set A nucleotide database using BLASTN (v2.5.0). BLAST searches were performed with an E-value threshold of ≤1 × 10^−5^, and only the top-ranked target hit for each query was retained (-max_target_seqs 1). Thus, each query sequence contributed at most one retained ARG annotation, while distinct query sequences within the same genome were evaluated independently. BLASTP and BLASTN searches were performed according to the sequence type of the reference databases and query files, with minimum identity and coverage thresholds set to 90% and 80%, respectively [30,31].

In this study, ARGs were defined as resistance-associated genetic determinants identified by sequence similarity to entries in the combined SARG and CARD reference database under the above criteria. Resistance-associated determinants were interpreted according to their established biological context and were not considered evidence of phenotypically confirmed AMR. ARG abundance was defined as the number of retained ARG annotations per genome. The identified ARGs were further classified according to antibiotic classes, and both ARG and VF profiles were summarized for each genome. For functional-category percentage calculations, retained ARG or VF annotation entries were pooled across all analyzed genomes. The proportion of each ARG or VF category was calculated as the number of retained annotation entries assigned to that category divided by the total number of retained ARG or VF annotation entries, respectively. Multilocus sequence typing (MLST) was performed using MLST v2.16.1 (https://github.com/tseemann/mlst, accessed on 21 September 2026) to determine the sequence types (STs) of the analyzed isolates [32].

### 2.3. ARG Risk Classification and Association with Mobile Genetic Elements

To evaluate the potential health risk of the resistome in *Brucella* spp., identified ARGs were classified into risk levels I to IV according to the predefined risk-ranking annotations provided in the SARG database [27]. The adopted framework considers human-associated enrichment, gene mobility, and host pathogenicity, with Rank I representing ARGs meeting all three criteria and therefore the highest risk category [27]. The distribution of ARG risk levels was then compared among different host sources, geographical regions, and time periods. To explore the potential association between ARGs and MGEs, MGE-related genes were identified using mobileOG-db [33]. MGE-associated gene abundance was defined as the number of MGE-related gene annotations detected in each genome and was not interpreted as the number of complete MGEs. Integrons and integron-associated ARGs were detected and annotated using IntegronFinder [34]. The co-occurrence of ARGs and MGE-associated genes within the same assembled contigs was further summarized to characterize potential genomic associations between ARGs and MGE-associated genes in *Brucella* spp.

### 2.4. Pan-Genome Construction and Functional Analysis

Prokka v1.14.5 was used to predict and annotate coding sequences in all 1059 genomes [35]. Subsequently, pan-genome analysis was performed using Roary (v3.13.0) [36] with the parameters -i 80 -e --mafft [37]. All 1059 genomes were included in the pan-genome analysis. The 80% identity threshold was selected based on preliminary exploratory analyses. An initial analysis using a 90% threshold did not yield a usable core-gene alignment for the complete multi-species dataset. The threshold was therefore reduced to 80% for the final pan-genome analysis. Core genes were defined as gene clusters present in 99–100% of the analyzed genomes according to the Roary output. Roary automatically handled gene clustering and paralogous gene assignment during pan-genome construction. Genes not meeting this criterion were considered accessory genes for subsequent analyses. Pan-genome openness was further quantitatively evaluated using a power-law accumulation model fitted to the mean pan-genome accumulation data generated from the Roary output. To explore the functional composition of accessory genes, accessory gene sequences were annotated using KofamKOALA v1.3.0 [38], and KEGG Orthology terms and related metabolic pathways were assigned.

### 2.5. Phylogenetic, Statistical Analyses and Visualization

For the dominant ST8 and ST11 lineages, core-gene alignments were generated separately from the corresponding genomes. The ST8 and ST11 core-gene alignments contained 2417 and 3089 core genes, respectively. The alignment lengths, numbers of variable sites, and parsimony-informative sites used for phylogenetic reconstruction are summarized in Table S10. Maximum-likelihood (ML) phylogenetic trees were then constructed using RAxML v8.2.13 [39] under the GTR + GAMMA model with 1000 bootstrap replicates. Because the phylogenetic analyses were intended to characterize broad within-lineage relationships rather than infer fine-scale transmission or recombination-adjusted evolutionary rates, no dedicated recombination filtering was applied. No additional alignment trimming or manual removal of ambiguous regions was performed. The resulting phylogenetic trees were used to explore the evolutionary relationships within the two dominant lineages. Temporal signal was assessed using TempEst (v1.5.3) based on root-to-tip regression between genetic distance and sampling year. The phylogenetic trees were annotated and visualized using iTOL v6.9 (https://itol.embl.de/, accessed on 21 September 2026) [40].

All statistical analyses were performed in R v4.4.3 unless otherwise specified. For continuous variables, two-sided pairwise comparisons between groups were performed using the Mann–Whitney U test. For gene carriage analysis, gene abundance values were converted into binary presence/absence data, and differences in carriage rates among groups were assessed using Fisher’s exact test based on group-by-presence/absence contingency tables. For multiple pairwise comparisons and gene-level Fisher’s exact tests, *p* values were adjusted using the false discovery rate (FDR) method, with adjusted *p* < 0.05 considered statistically significant. Statistical significance was indicated as follows: * *p* < 0.05, ** *p* < 0.01, *** *p* < 0.001. Associations among ARG abundance, VF abundance, and MGE-associated gene abundance were assessed using Spearman rank correlation. For β-diversity analysis, Bray–Curtis dissimilarities were used for principal coordinates analysis (PCoA), and differences among groups were assessed using permutational multivariate analysis of variance (PERMANOVA) with 999 permutations in the vegan package. Multivariate dispersion was assessed using the betadisper function followed by permutest with 999 permutations. *p* values from the prespecified PERMANOVA comparisons across host, continental, and temporal factors were adjusted using the Benjamini–Hochberg false discovery rate (FDR) method.

## 3. Results and Discussion

### 3.1. Geographical Distribution of the Global Brucella spp. Genomes

A total of 1760 publicly available *Brucella* spp. genomes were retrieved worldwide, of which 1059 met the inclusion criteria and were used for further analyses (Figure 1A). These genomes had complete information on source, host, isolation date, and country/region. In this study, isolation sources were classified into four categories: human, livestock, wildlife and environmental sources. Livestock-derived isolates accounted for the largest proportion (46.4%, *n* = 491), followed by human (39.1%, *n* = 414), wildlife (9.7%, *n* = 103), and environmental isolates (4.8%, *n* = 51) (Figure 1B). The livestock-derived *Brucella* spp. strains were predominantly isolated from cattle (36.0%), followed by goat/sheep (25.3%) and swine (1.4%). Among human samples, blood constituted the dominant source (71.3%), whereas the remaining strains were derived from other human-associated (19.2%) or unclassified clinical samples (9.5%). Wildlife-derived strains were mainly recorded as other or unclassified sources (67.0%), followed by tissue samples (17.5%). Environmental isolates were mainly obtained from river/wastewater (31.4%) and soil (29.4%) (Figure S1A).

Geographically, the isolates were mainly derived from Asia (*n* = 441, 41.6%), Europe (*n* = 376, 35.5%) and Africa (*n* = 94, 8.8%) (Figure 1D). Among 74 countries/regions, China contributed the largest proportion of genomes (20.3%), followed by Italy (16.6%) and India (8.7%). This uneven geographic representation may partly reflect differences in brucellosis burden, surveillance intensity, and sequencing or data-submission efforts among countries [41]. On the timescale, the genomes were divided into 1940–1965 (*n* = 37, 3.5%), 1966–1990 (*n* = 73, 6.9%), 1991–2015 (*n* = 520, 49.1%), and 2016–2025 (*n* = 429, 40.5%) in chronological order (Figure 1C; Figure S1C). The marked differences in genomic sampling intensity across these periods likely reflect variations in sample availability, surveillance intensity, and sequencing efforts over time, which should be considered when interpreting temporal patterns.

Among the 1059 retained genomes, 836 genomes (78.9%) were assigned to 49 defined STs, whereas 223 genomes (21.1%) remained unclassified based on the available MLST profiles. Examination of the unclassified profiles showed that these genomes included those with missing or unresolved MLST loci, partial or uncertain allele matches, and putative novel alleles. In addition, some genomes had complete allele profiles, but their allele combinations did not correspond to any currently defined ST in the PubMLST database. These profiles were therefore retained as unclassified and were not designated as novel STs.

ST8 (28.2%) and ST11 (13.1%) were identified as the predominant STs. ST8 was predominantly represented among human-derived isolates and in Asia, whereas ST11 was more frequently represented among livestock-derived isolates and in Europe (Figure 1E–G). In the temporal analysis, ST2 and ST1 were the main genotypes before 1990. After 1991, ST8 and ST11 became more frequently represented and were the predominant STs in the dataset (Figure 1H). The UpSet analysis further showed that most STs were continent-specific, with Europe, Asia, and Africa containing 17, 7, and 5 unique STs, respectively (Figure S1B). Previous studies have shown that dominant *Brucella* genotypes vary geographically and exhibit clear regional characteristics [42]. The wide distribution of ST8 and ST11 in Eurasia may be associated with frequent animal movement and animal-product exchange across this region [43,44] (Figure 1I).

### 3.2. Host-Associated Variation in Resistome Profiles and Risk Characteristics of Brucella spp.

To characterize the resistome composition and potential dissemination risk of *Brucella* spp., ARGs were annotated using the SARG [27] and CARD [28] databases. A total of 8 ARG types and 34 ARG subtypes were identified in *Brucella* spp. genome dataset. Peptide antibiotic resistance accounted for 91.6% of the pooled ARG annotations, followed by β-lactam (4.3%), aminoglycoside (2.6%), and phenicol resistance (0.5%). The four categories contained 2, 13, 13, and 1 detected ARG subtypes, respectively. The dominance of the peptide category was mainly attributable to the widespread presence of *Bsui_mprF* and *bacA*. Their widespread occurrence likely reflects conserved intrinsic resistance-associated mechanisms in *Brucella* spp. rather than a high burden of clinically relevant AMR determinants [45,46,47,48].

We further compared ARG abundance, resistome composition, and risk characteristics across isolation sources, continents, and time periods (Table S2). Environmental isolates showed significantly higher ARG abundance per genome than other source groups (median: 3 ARGs per genome vs. 2 ARGs per genome in human and livestock isolates; *p* < 0.001), whereas human and livestock isolates generally displayed relatively lower ARG burdens (Figure 2A). This result suggests a greater representation of ARGs among environmental isolates, consistent with the role of wastewater, soil, and sediments as potential reservoirs receiving inputs from human activities and livestock production [49]. Nevertheless, differences in species composition, genome and assembly characteristics, sequencing background, geographic origin, and sampling period may also partly contribute to the observed variation among source groups. Across continents, ARG abundance was broadly comparable among regions, with all continental groups showing the same median value of 2 ARGs per genome. Notably, several Asian isolates showed relatively high ARG counts, reaching up to 14 ARGs per genome (Figure 2B). Temporally, although all isolation periods showed the same median ARG abundance of 2 ARGs per genome, genomes isolated during 2016–2025 displayed a broader distribution of ARG abundance than those from earlier periods (*p* < 0.01; Figure 2C). ARG class composition also showed increased relative proportions of ARGs associated with β-lactam and aminoglycoside resistance in the 2016–2025 group (Figure S2).

Then, the Shannon diversity of ARGs was calculated to evaluate the α-diversity of resistome profiles. The α-diversity trends were generally similar to those observed for ARG abundance across host, continental and temporal groups. Notably, Shannon diversity varied among continental groups, whereas ARG abundance showed limited variation at the continental level (*p* < 0.05; Figure S3). To further assess differences in ARG composition among groups, PCoA was performed based on ARG profiles. From the host perspective, PERMANOVA indicated significant differences in ARG composition among host-associated groups (PERMANOVA *p* = 0.001, FDR-adjusted *p* = 0.0012, R^2^ = 0.104). Environmental isolates showed a relatively distinct distribution from human, livestock, and wildlife isolates (Figure 2D). At the continental level, ARG profiles also differed among regions (PERMANOVA *p* = 0.001, FDR-adjusted *p* = 0.0012, R^2^ = 0.03). However, the small effect size indicated that continental grouping explained only a limited proportion of the overall resistome variation. Most continental groups showed substantial overlap in the PCoA space, with Asian isolates exhibiting partial separation from isolates from Africa, Europe, and North America (Figure 2E). Temporal comparisons showed weaker but detectable differences in ARG composition among time periods (PERMANOVA *p* = 0.022, FDR-adjusted *p* = 0.022, R^2^ = 0.007), with sampling period explaining only a very small proportion of the overall variation. Considerable overlap remained among time groups, although slight shifts in distribution were observed across different periods (Figure 2F). Together, these results indicate that resistome composition varied among the examined groups. Source grouping showed a larger effect size than geographic and temporal grouping, although the overall explained variation remained limited.

The potential resistance risk of ARGs carried by *Brucella* spp. was subsequently evaluated using risk-rank classification. As shown in Figure 2G, we found that the majority of genomes were categorized as Rank IV ARG carriers. Nevertheless, several Rank I in the SARG database [27] were detected, including *floR*, *AAC(6′)-Ib9*, *AAC(3)-II*, *aadA-ANT(3″)-IIa*, *mphF*, and *APH(6)-Id*. These genes were mainly associated with resistance to aminoglycosides, macrolides, and phenicols, suggesting that clinically relevant resistance determinants were present in *Brucella* genomes.

Source-based analysis further showed that isolates carrying Rank I high-risk ARGs were mainly derived from environmental (63.64%) and human (36.36%) sources and were primarily distributed in China, Pakistan, and India. The observed distribution of these Rank I ARGs is consistent with possible ecological links among livestock production, environmental contamination, and human exposure. Environmental matrices such as wastewater, soil, and sediments can receive antibiotic residues, antibiotic-resistant bacteria (ARB), and ARGs from livestock and human activities [50]. These environments may therefore serve as potential reservoirs and ecological interfaces for resistance determinants. Meanwhile, the detection of Rank I ARGs in human-derived isolates may be associated with exposure to infected animals or contaminated environments [51]. Antimicrobial treatment pressure during clinical infection may also influence the occurrence and distribution of clinically relevant resistance determinants [52]. These findings highlight the need to monitor ARG profiles in environmental and human-derived *Brucella* isolates, especially high-risk and clinically relevant ARGs, to improve the assessment of resistance risks.

### 3.3. Spatiotemporal Variation and Species-Specific Distribution of Virulence Determinants in Brucella spp.

In this study, a total of 55 distinct virulence factor genes were detected in the *Brucella* spp. genomic dataset. Among the pooled VF annotation entries across all analyzed genomes, LPS-associated factors accounted for 43.3% of the total annotations, followed by T4SS-secreted effectors (20.8%) (Figure S4). The predominance of these two categories indicates that the predicted VF profile was mainly associated with immune modulation and intracellular trafficking [22]. This agrees with previous descriptions of *Brucella* spp. as intracellular pathogens that generally lack classical extracellular virulence factors, such as exotoxins, cytolysins, and extracellular degradative enzymes. Instead, their pathogenicity mainly relies on host interaction and intracellular survival mechanisms [53].

To compare VF profiles across different scales, VF abundance, α-diversity, and β-diversity were analyzed according to host source, geographic region, and time period (Table S3). Significant differences in VF abundance were observed among different source groups (Figure 3A). In contrast to the ARG results, environmental isolates showed the lowest VF abundance, whereas human-derived isolates showed the highest VF abundance (median: 12 VFs per genome), followed by livestock-derived isolates (median: 9 VFs per genome; *p* < 0.01). Although some livestock-derived genomes carried up to 36 VFs per genome, the overall distribution suggested higher VF abundance in human-derived isolates. However, these source-associated differences may also be partly influenced by variation in species composition, ST distribution, geographic representation, and sampling period among the source groups. Across continents, VF abundance was relatively higher in isolates from Africa, Asia, and Europe than in those from North America, Oceania, and South America, with African isolates showing the highest median VF count per genome (median: 13 VFs per genome; *p* < 0.05) (Figure 3B). The observed geographic differences may also be influenced by variation in species composition, sampling intensity, and other characteristics of the available genomic dataset. Temporally, VF abundance varied among isolation periods, but the median values did not show a consistent increasing or decreasing trend (Figure 3C).

A similar pattern was observed for VF α-diversity. The Shannon index differed among source groups, with the lowest values observed in environmental isolates. Within host-associated isolates, human and livestock sources showed similar diversity levels. At the continental scale, isolates from Africa, Asia, and Europe exhibited relatively higher Shannon index values than those from other continents (Figure S3). Across isolation periods, the Shannon index varied among groups but did not show a consistent increasing or decreasing trend, which was generally consistent with the temporal pattern of VF abundance. The higher VF abundance and diversity in host-associated isolates, especially those from human and livestock sources, may reflect the importance of virulence-related functions during *Brucella* infection. This is supported by previous studies showing that the VirB type IV secretion system, LPS biosynthesis-related factors, and outer membrane proteins contribute to intracellular survival, immune modulation, and host–pathogen interactions [54].

β-diversity analysis was further performed to evaluate differences in VF composition among groups. PCoA showed partial separation among isolates from different host sources, although substantial overlap was still observed (PERMANOVA *p* = 0.001, FDR-adjusted *p* = 0.0012, R^2^ = 0.095) (Figure 3D). Multivariate dispersion analysis indicated significant differences in within-group dispersion among host groups (permutest, *p* = 0.001), suggesting that dispersion differences may have contributed to the observed host-associated pattern (Table S4). Isolates from livestock and human sources were mainly concentrated near the center of the ordination space, whereas isolates from wildlife sources appeared more broadly distributed. At the continental scale, isolates from Asia, Europe, and Africa largely overlapped, whereas isolates from Oceania showed a tendency to separate from the other groups (PERMANOVA *p* = 0.001, FDR-adjusted *p* = 0.0012, R^2^ = 0.090) (Figure 3E). For different isolation periods, most groups overlapped extensively and did not form clearly independent clusters (PERMANOVA *p* = 0.001, FDR-adjusted *p* = 0.0012, R^2^ = 0.013) (Figure 3F). Although a statistically significant temporal difference was detected, sampling period explained only 1.3% of the overall variation in VF composition, indicating a very limited temporal effect. PERMANOVA showed significant differences among host sources, continents, and time periods, although the relatively low R^2^ values indicated that these factors explained only a limited proportion of the overall variation in VF composition. Notably, multivariate dispersion analysis suggested that within-group variability may have contributed to the host-associated differences observed in VF profiles. Together with the extensive overlap observed in PCoA space, these results indicate that the virulence repertoire of *Brucella* spp. remained broadly conserved, while host-associated differences may also have been influenced by differences in within-group variability. Gene-level enrichment analysis further indicated that several ARGs and VFs exhibited uneven distributions across source, geographic, and temporal categories, suggesting that group-associated variation may also be reflected at the individual gene level (Figure S5).

Among *Brucella* spp., *B. melitensis*, *B. abortus*, and *B. suis* are considered the major pathogenic species with important implications for human and animal health [55]. Given their epidemiological and pathogenic importance, ARG and VF profiles were further compared among these three species in this study. Consistent with the presence/absence profiles shown in Supplementary Figure S6, Fisher’s exact test with FDR correction identified species-associated enrichment of selected ARGs and VFs, indicating that some resistance and virulence determinants showed uneven prevalence among *B. melitensis* (*n* = 505), *B. abortus* (*n* = 319), and *B. suis* (*n* = 37). Among the detected ARGs, *B. melitensis* showed a higher prevalence of several ARGs associated with aminoglycoside and β-lactam resistance than *B. abortus* and *B. suis* (Figure 4A). In contrast, VF profiles were more broadly conserved across the three major pathogenic species. Several virulence factor genes, including those involved in immune modulation, LPS biosynthesis, outer membrane-associated adhesion, and the type IV secretion system, were detected across multiple species rather than being restricted to a single species. Nevertheless, some VF genes showed different prevalence patterns among the three species, suggesting that the virulence repertoire was conserved overall but varied in specific genes. These observations indicate that species-level differences were more evident for selected ARGs than for the major VF repertoire. This may reflect the relatively conserved intracellular pathogenic strategy of *Brucella* spp., whereas these resistance-associated genes showed greater variability among species and may represent important features for antimicrobial resistance surveillance [54,56].

### 3.4. Global Pan-Genomic Architecture of Brucella spp.

To further characterize the genomic diversity and pan-genome structure of the global *Brucella* spp. collection, we performed pan-genome analysis. Across all 1059 genomes, a total of 69,871 gene clusters were identified, including 360 core, 469 soft-core, 3256 shell, and 65,786 cloud gene clusters. Accordingly, the accessory genome, defined as all non-core gene clusters, comprised 69,511 gene clusters. The pan-genome curve showed a continuous increase in gene number as additional genomes were included (Figure 4B). Quantitative fitting of the pan-genome accumulation data yielded an accumulation parameter of γ = 0.5033 (95% CI: 0.5018–0.5048; R^2^ = 0.9975). This result provided quantitative support for continued pan-genome expansion and an open pan-genome structure. Source-stratified analyses also showed differences in pan-genome accumulation patterns among environmental, human, livestock, and wildlife isolates. The fitted accumulation parameters were relatively higher for wildlife and environmental isolates than for human and livestock isolates (Table S5). Across geographical and temporal groupings, the pan-genome profiles showed limited divergence, suggesting that *Brucella* spp. remained broadly conserved at the pan-genome level across continents and isolation periods (Figure S7). Consistent with our findings, a recent pan-genome-wide association and machine-learning study based on 991 *Brucella* genomes also reported an open pan-genome for *Brucella* spp. and suggested that host origin may influence the predicted zoonotic potential of closely related *Brucella* strains [57].

To further evaluate source-associated differences in conserved gene sets, core-genome sets were independently defined within each source group and their overlap was subsequently compared. Comparison of these within-source core-genome sets showed that 61.0% and 56.1% of the core gene clusters identified within the human and livestock groups, respectively, were unique to their corresponding source-group core sets. The remaining gene clusters were shared with at least one other source group (Figure 4C). These results indicate differences in the composition and overlap of within-source core-genome sets among isolation sources.

Because accessory genes represent the variable component of bacterial pan-genomes, we further performed KEGG functional annotation of all accessory genes to assess their potential functional profiles [58]. Accessory genes were mainly annotated to signaling and cellular processes (33.8%), genetic information processing (16.6%), amino acid metabolism (4.4%), and carbohydrate metabolism (3.2%) (Figure 4D). UpSet analysis further showed substantial sharing of accessory gene clusters among different isolation-source groups (Figure 4E). These results describe the major KEGG functional categories represented in the accessory gene pool. Overall, the pan-genome analysis revealed source-related differences in gene accumulation patterns, core gene composition, and accessory gene functional profiles.

### 3.5. Phylogenetic Structure and Spatiotemporal Clustering of Dominant Brucella Lineages (ST8 and ST11)

Based on the MLST results, 836 strains of the 1059 strains of *Brucella* spp. were assigned to 49 STs. ST8 (*n* = 299, 28.2%) and ST11 (*n* = 139, 13.1%) were the predominant STs, together accounting for 41.4% of the complete dataset. ST8 was predominantly represented among human-derived isolates and was mainly distributed in Asia (*n* = 254, 84.9%), whereas ST11 was more frequently represented among livestock-derived isolates and was mainly distributed in Europe (*n* = 99, 71.2%). To further investigate the phylogenetic relationships within these two dominant lineages, maximum-likelihood phylogenetic trees were constructed for ST8 and ST11 based on core genome alignments. SNP-sites analysis further identified sequence variations within these two lineages, providing additional evidence of genetic diversity among ST8 and ST11 isolates (Tables S6 and S7).

In the ST8 phylogeny, isolates from the same or neighboring geographical regions tended to cluster within closely related branches, whereas isolates from different continents were distributed across distinct parts of the tree. Human and livestock isolates were frequently observed within the same clades. Several Asian isolates also clustered within closely related branches regardless of source background (Figure 5A). Similar geographically structured patterns have been reported in previous studies [43,44]. A similar pattern was observed in the ST11 phylogeny, where isolates showed partial clustering by continental origin, while human- and livestock-derived isolates were frequently intermingled within the same clades (Figure 5B). These observations suggest that geographical origin may be associated with the phylogenetic distribution of ST8 and ST11 isolates, although source-associated patterns were also observed.

The distribution of sampling years across the ML trees showed that recent isolates (2016–2025) were often located on terminal branches within established regional clades. In several cases, recently isolated strains clustered with earlier isolates from the same geographical region, indicating temporal overlap among genetically related regional lineages in the available dataset. However, sampling was non-random and unevenly distributed across isolation periods, and these patterns may therefore partly reflect differences in genome availability and sampling intensity over time. Accordingly, the temporal patterns were interpreted descriptively and were not used to infer lineage persistence, divergence times, or evolutionary rates.

To assess whether molecular dating could be reliably performed, temporal signal was evaluated using root-to-tip regression in TempEst (v1.5.3). The correlation between genetic divergence and sampling time was weak and variable, with correlation coefficients ranging from 0.0319 to 0.4434 (Figure S8). Previous studies have also reported weak or limited temporal signal in modern *Brucella* genome datasets, possibly due to slow evolutionary rates and lineage-specific differences in mutation accumulation [59]. Although Bayesian molecular dating was explored, the weak temporal signal indicated that the resulting time-calibrated estimates were not sufficiently reliable for biological interpretation. Therefore, estimates of divergence times and evolutionary rates were not used in the main analysis. The temporal patterns described above were based on the distribution of sampling dates across the ML phylogenies rather than molecular clock-based dating.

### 3.6. MGE-Associated Genes and Their Genomic Associations with ARGs in Brucella spp.

To characterize MGE-associated genes and their relationships with ARGs and VFs in *Brucella* spp., we performed annotation and co-occurrence analyses of MGE-associated genes. Considerable variation in MGE-associated gene abundance was observed among strains. Figure 6A illustrates the joint distribution of ARG abundance, VF abundance, and MGE-associated gene abundance across isolates from different sources. Environmental isolates tended to occur in regions with higher MGE-associated gene abundance, whereas human and livestock isolates were mainly distributed at lower MGE-associated gene abundance, with substantial overlap among source groups. These values represent annotated MGE-associated genes rather than complete mobile genetic elements. Overall, MGE-associated gene profiles varied markedly among strains.

MGE-associated gene profiles differed among source, continent, and temporal groups, but substantial overlap in the PCoA plots indicated that these differences did not represent complete group separation (Figure S9). From the isolation source perspective, environmental and wildlife isolates generally harbored higher MGE-associated gene abundance than human and livestock isolates, whereas human, livestock, and wildlife isolates tended to exhibit relatively higher MGE-associated gene diversity than environmental isolates. At the continental level, isolates from Oceania showed comparatively higher MGE-associated gene abundance and diversity, while differences among the remaining continents were relatively limited. Across different isolation periods, MGE-associated gene profiles varied only modestly, with no consistent temporal trend observed. Overall, source-associated differences were more apparent in the present dataset than geographic or temporal differences. However, these patterns represent associations and may also be influenced by differences in species and ST composition among groups.

Functional classification showed that MGE-associated genes were predominantly annotated to integration and excision (70.2%), followed by phage-related functions (20.2%), transfer-related functions (6.4%), stability/transfer/defense functions (1.9%), and replication/recombination/repair functions (1.3%) (Figure 6E). These results describe the major functional categories represented among the detected MGE-associated genes.

Spearman rank correlation analysis revealed different patterns of association among ARG abundance, VF abundance, and MGE-associated gene abundance. ARG abundance showed a weak negative correlation with VF abundance (Spearman’s ρ = −0.288, *p* < 0.001; Figure 6B), whereas no significant correlation was detected between ARG abundance and MGE-associated gene abundance (ρ = 0.014, *p* = 0.661; Figure 6C). VF abundance showed a moderate negative correlation with MGE-associated gene abundance (ρ = −0.462, *p* < 0.001; Figure 6D). These correlations describe genome-level associations among the three annotation categories and should not be interpreted as evidence of direct mechanistic relationships. Stratified sensitivity analyses by major species and ST groups were further performed to evaluate the influence of population structure (Figure S10; Table S8). The VF–MGE association remained consistent across major groups after FDR correction, whereas ARG-related associations were less consistent. Although the stratified sensitivity analyses considered major population structure at the species and ST levels, residual confounding from finer scale phylogenetic background and genome composition cannot be excluded. Previous studies in other bacterial species have reported associations between MGEs, variable genomic regions, and antimicrobial resistance determinants [60]. The biological basis of the associations observed in the present dataset remains unclear. Previous studies have proposed that factors such as fitness costs, genome stability, and niche-specific selection may influence the retention or loss of accessory genes and adaptive determinants [61]. However, whether these factors contribute to the patterns observed here cannot be determined from the present analysis.

We further investigated the genomic co-occurrence of ARGs and MGE-associated genes by identifying their occurrence on the same contigs (Table S9). In this analysis, co-occurrence refers to the presence of ARGs and MGE-associated genes on the same assembled contig rather than proximity within a predefined genomic distance threshold. Gene coordinates, order, strand orientation, and intervening annotated genes were retained when describing the local genomic organization. *Brucella* spp. may circulate in livestock production systems where antibiotics are used for the prevention and treatment of bacterial infections [7]. Given this background and the importance of antimicrobial therapy for human brucellosis, particular attention was given to ARGs associated with antimicrobial classes relevant to both animal production and human treatment [13]. The detected ARGs were mainly associated with aminoglycoside and β-lactam resistance, two antimicrobial classes with relevance to both animal production systems and human brucellosis treatment. Several selected ARG containing contigs also contained genes involved in integration, excision, transposition, or DNA transfer. Figure 6F shows the genomic organization of these regions, including gene order and orientation. Among these ARGs, *VIM-4* and *aadA-ANT(3″)-IIa* were selected for detailed analysis because they represent different antimicrobial resistance classes and were detected in selected contigs containing multiple MGE-associated gene annotations.

Three environmental isolates contained *VIM-4* on contigs NZ_RHTD01000025.1, NZ_JAODZT010000029.1, and NZ_JAYKZA010000005.1. The first two contigs showed a similar local gene organization involving *qacEdelta1*, multiple attC sites, *aadA-ANT(3″)-IIa*, *dfrA1*, *aac(6′)-Il*, and *VIM-4*. In both regions, these genes occurred in a similar order, with multiple attC sites interspersed among the resistance determinants. In contrast, the third *VIM-4* containing contig showed a different local arrangement involving *intI1*, *AAC(6′)-Ib7*, attC sites, *VIM-4*, *qacEdelta1*, and *sul1*. IntegronFinder identified integron associated structures in these regions, and the detailed gene organization, genomic coordinates, and orientations are provided in Table S9. These genomic features support an integron associated context for *VIM-4*, but do not demonstrate integron activity or active horizontal transfer. Among the three VIM-4 containing contigs, two environmental contigs, NZ_RHTD01000025.1 and NZ_JAODZT010000029.1, also carried *aadA-ANT(3″)-IIa*, an aminoglycoside resistance gene. Additional ARG and MGE-associated genomic arrangements are provided in Supplementary Figure S11. Notably, several ARGs identified as high-risk determinants in this study, including *floR*, *AAC(3)-II*, *AAC(6′)-Ib9*, and *aadA-ANT(3″)-IIa*, were also detected in the same contig genomic context analysis. These genomic contexts suggest potential mobility of the ARG containing regions, but do not demonstrate active horizontal transfer.

Across selected ARG containing contigs, the transposase gene *tnpA*, the integrase gene *intI1*, and several genes associated with DNA transfer were also detected. These included *traA*, *traG*, *trbC*, *trbE*, *trbF*, and *trbL*. These genes are associated with DNA integration, recombination, or transfer functions [62]. However, their presence in ARG-containing regions does not demonstrate a complete or functional conjugative system and does not provide evidence of active horizontal transfer. Similar One Health studies have suggested that environmental compartments can contribute to the maintenance and circulation of AMR-associated genetic elements among human, animal, and environmental sources [63]. Because the present evidence is based on genomic annotation and co-occurrence within assembled contigs, it cannot determine whether these regions are functionally mobile or whether horizontal transfer has occurred. Additional comparative genomic and experimental evidence would be required to establish such processes. The occurrence of ARGs relevant to human treatment and animal production within genomic regions containing MGE-associated genes supports continued surveillance of ARG/MGE associations at the human, animal, and environmental interface.

To assess the robustness of the principal genomic findings to assembly quality, all major analyses were additionally evaluated using the 800-genome HQ subset. The dominant ARG and VF profiles, the open pan-genome interpretation, and the broad phylogenetic structure were retained after quality filtering; however, rare ARGs, accessory/pan-genome features, and particularly MGE-associated annotations showed quantitative sensitivity to genome quality. Detailed methods, statistical comparisons, and results are provided in Supplementary Figures S12–S15 and Table S12.

### 3.7. Limitations

Several limitations should be considered when interpreting the findings of this study. First, although genome-wide comparative analyses provide insights into the distribution of ARGs, VFs, and MGE-associated genes, the identification of MGE-associated genes does not represent the presence of complete mobile genetic elements. Similarly, the co-occurrence of ARGs with integrase, transposase, phage, or transfer-associated genes within assembled contigs indicates potential genomic associations but does not demonstrate active mobility or horizontal transfer.

Second, the public genomic datasets analyzed in this study were derived from heterogeneous sources with uneven distributions across species, STs, geographic regions, and sampling periods. Therefore, observed differences among source groups and genome-level associations may partly reflect underlying population structure and sampling composition. In addition, the inclusion of genomes with complete metadata may introduce selection bias because genomes with incomplete metadata were excluded. The analyses also depend on existing database annotations and may therefore be affected by annotation bias. Although no genome-quality thresholds were applied to define the primary 1059-genome dataset, the additional sensitivity analysis using 800 genomes with completeness ≥95% and contamination ≤5% showed that the principal ARG, VF, pan-genome, and phylogenetic patterns were broadly retained after quality filtering. However, the sensitivity analysis also indicated that some quantitative genomic features, particularly MGE-associated annotations, were affected by genome quality. In addition, completeness and contamination alone do not capture all aspects of assembly quality. Assembly fragmentation, contig number, N50, genome length, and local assembly errors were not incorporated as filtering criteria and may still influence gene detection and apparent gene absence. Therefore, residual effects of assembly quality, particularly for accessory and MGE-associated genomic features, cannot be completely excluded.

Third, the correlations among ARG, VF, and MGE-associated gene abundance represent statistical associations at the genome level and should not be interpreted as direct biological mechanisms. Additional phylogeny-aware analyses and experimental validation will be required to further clarify the evolutionary and functional relationships among these genomic features.

Finally, although temporal information was incorporated into the comparative analysis, the weak temporal signal observed in the dataset limits molecular-clock-based evolutionary inference. Therefore, the temporal patterns described in this study should be interpreted as changes in genomic distribution rather than direct estimates of evolutionary rates or divergence times.

## 4. Conclusions

This study provides a global genomic perspective on the population structure, genomic diversity, and AMR-related features of *Brucella* spp. Variation in dominant ST lineages, accessory genes, VFs, ARGs, and MGE-associated genes revealed differences among isolates from different host and ecological sources, although these patterns may be influenced by sampling composition and population structure. Pan-genome and phylogenetic analyses revealed an open but relatively conserved genomic structure, with environmental and wildlife isolates showing relatively broader gene repertoires than human and livestock isolates in the analyzed dataset. Selected high-risk ARGs were detected within genomic regions containing MGE-associated genes. These genomic contexts suggest potential mobility of ARG-associated regions, but do not demonstrate active horizontal transfer. These findings emphasize the value of genomic surveillance across human, livestock, wildlife, and environmental sources. They also support a One Health approach for monitoring the evolution and potential dissemination of AMR-related determinants in *Brucella* spp.

## Figures and Tables

**Figure 1:**
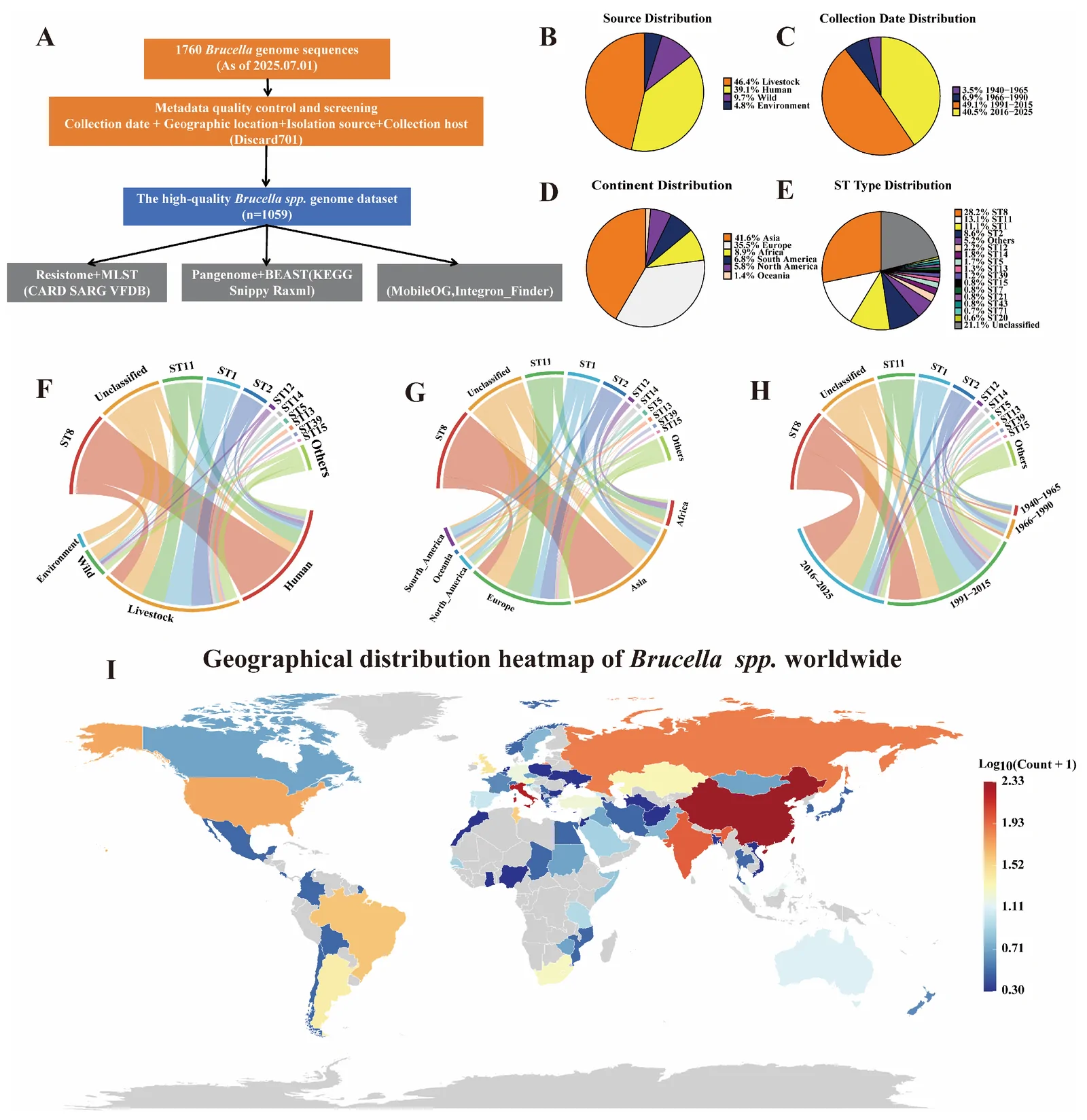
Global characteristics of *Brucella* spp. genomes. A total of 1760 genomes were initially retrieved, of which 1059 met the inclusion criteria and were used for the analyses shown in panels (**B**–**I**). (**A**) Flowchart of the global *Brucella* spp. genome analysis pipeline. (**B**) Distribution of *Brucella* spp. genomes from different isolation sources. (**C**) Distribution of *Brucella* spp. genomes across different sampling periods. (**D**) Distribution of *Brucella* spp. genomes across continents. (**E**) Top 15 STs in the global *Brucella* spp. genome dataset. (**F**–**H**) Chord diagrams showing the distribution of *Brucella* spp. STs by isolation source (**F**), geographical origin (**G**), and sampling period (**H**). (**I**) Global map showing the geographical distribution of 1059 *Brucella* spp. genomes from 74 countries/regions across six continents.

**Figure 2:**
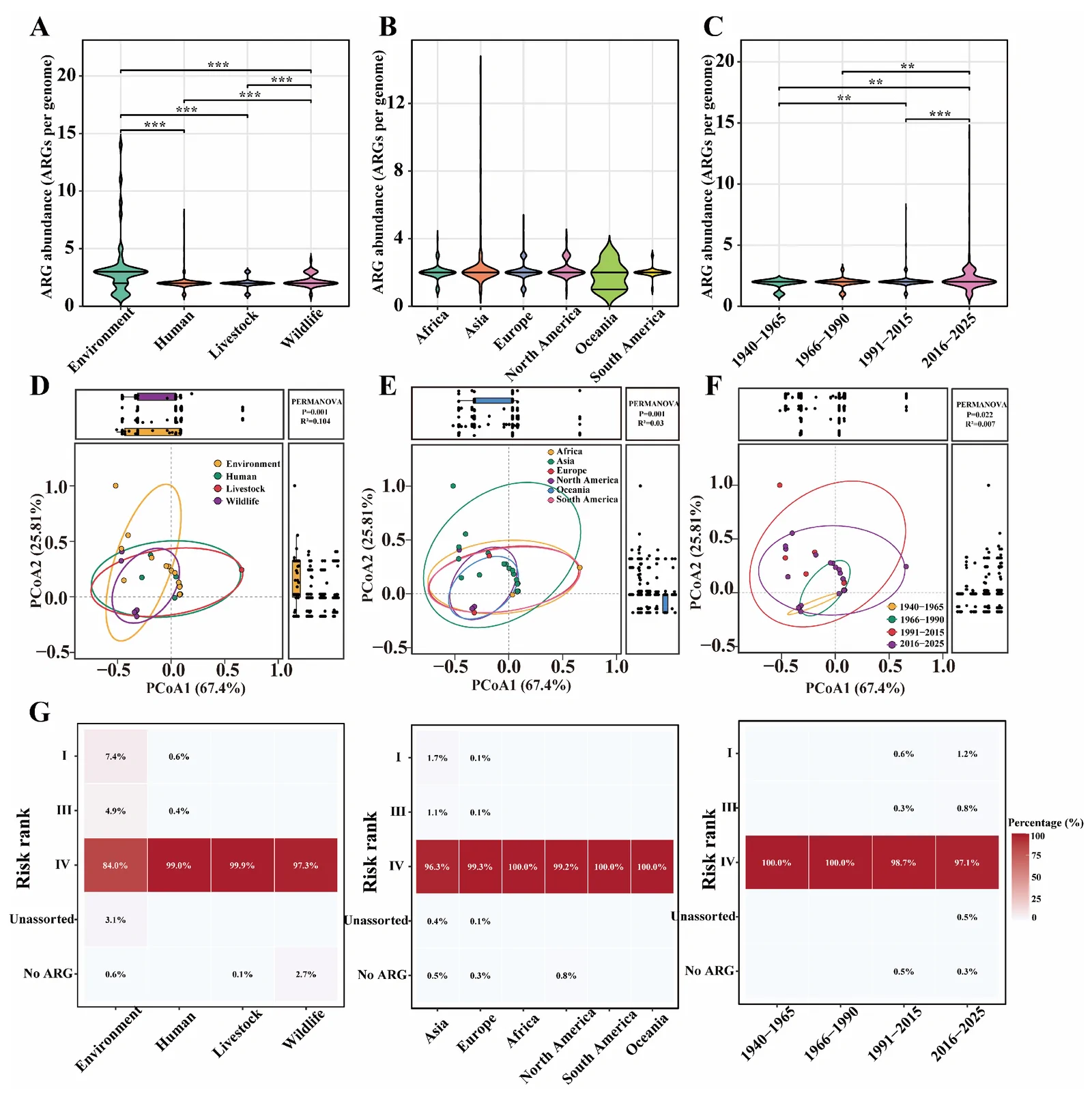
Distribution, composition, and risk classification of ARGs in *Brucella* spp. genomes. (**A**–**C**) Differences in ARG abundance, expressed as ARGs per genome, among isolates grouped by isolation source (**A**), continent (**B**), and isolation period (**C**). Violin plots show the distribution of ARG abundance in each group, with statistical significance indicated by horizontal brackets. (**D**–**F**) PCoA based on ARG profiles showing the distribution of resistome composition among isolates from different sources (**D**), continents (**E**), and isolation periods (**F**). Colored ellipses represent the 95% confidence regions of the corresponding groups in the PCoA space, and PERMANOVA results are shown in each panel. (**G**) Heatmaps showing the proportional distribution of ARG risk ranks across different sources, continents, and isolation periods. Color intensity represents the percentage of ARGs assigned to each risk rank. Statistical significance is indicated as ** *p* < 0.01 and *** *p* < 0.001.

**Figure 3:**
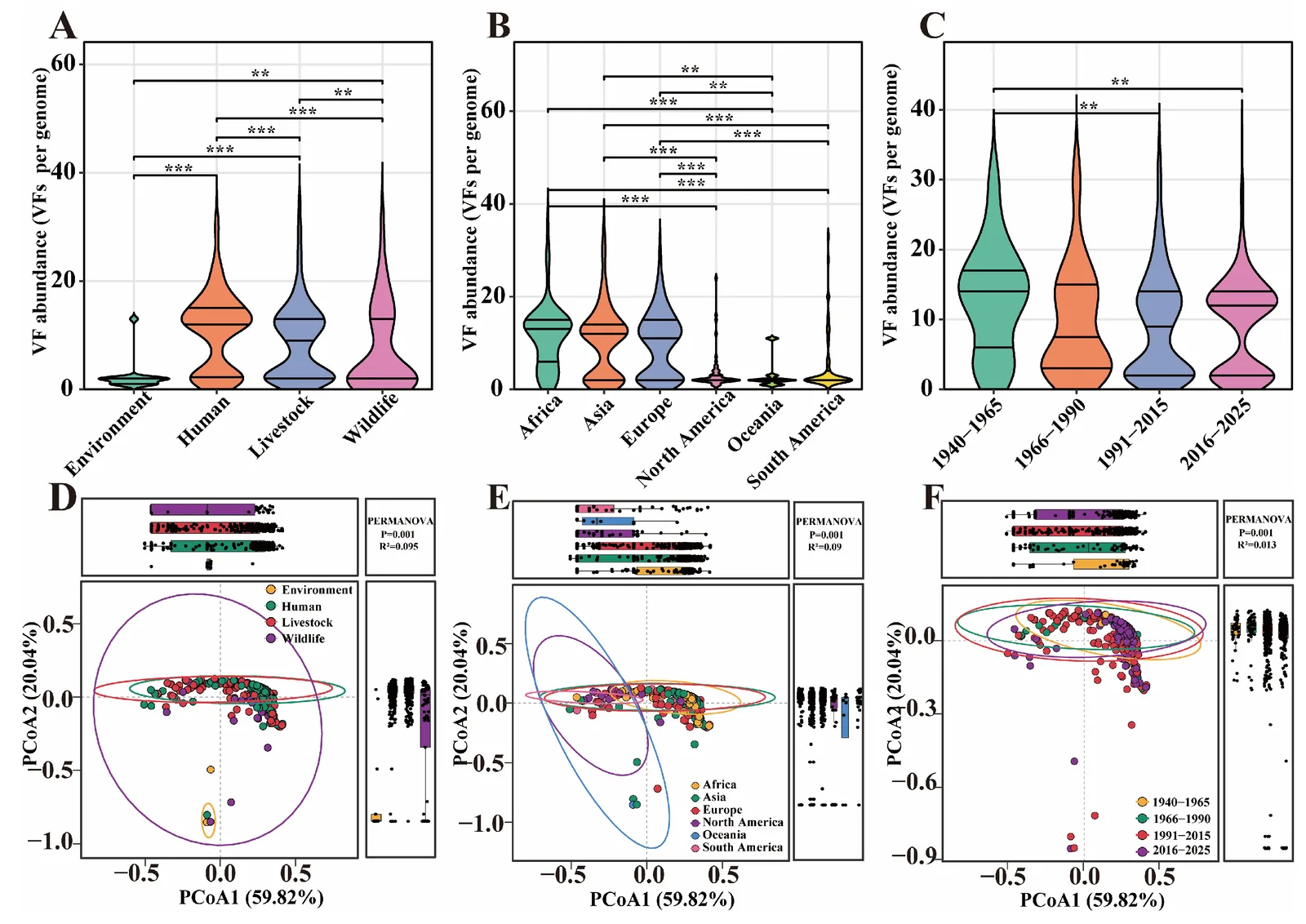
Variation in VF abundance and virulence-associated gene composition across source, geographic, and temporal groups. (**A**–**C**) VF abundance, expressed as VFs per genome, was compared among isolates from different sources (**A**), continents (**B**), and isolation periods (**C**). (**D**–**F**) PCoA based on VF profiles was used to evaluate differences in virulence-associated gene composition among source (**D**), continental (**E**), and temporal (**F**) groups, with PERMANOVA results shown in each panel. Statistical significance is indicated as ** *p* < 0.01 and *** *p* < 0.001.

**Figure 4:**
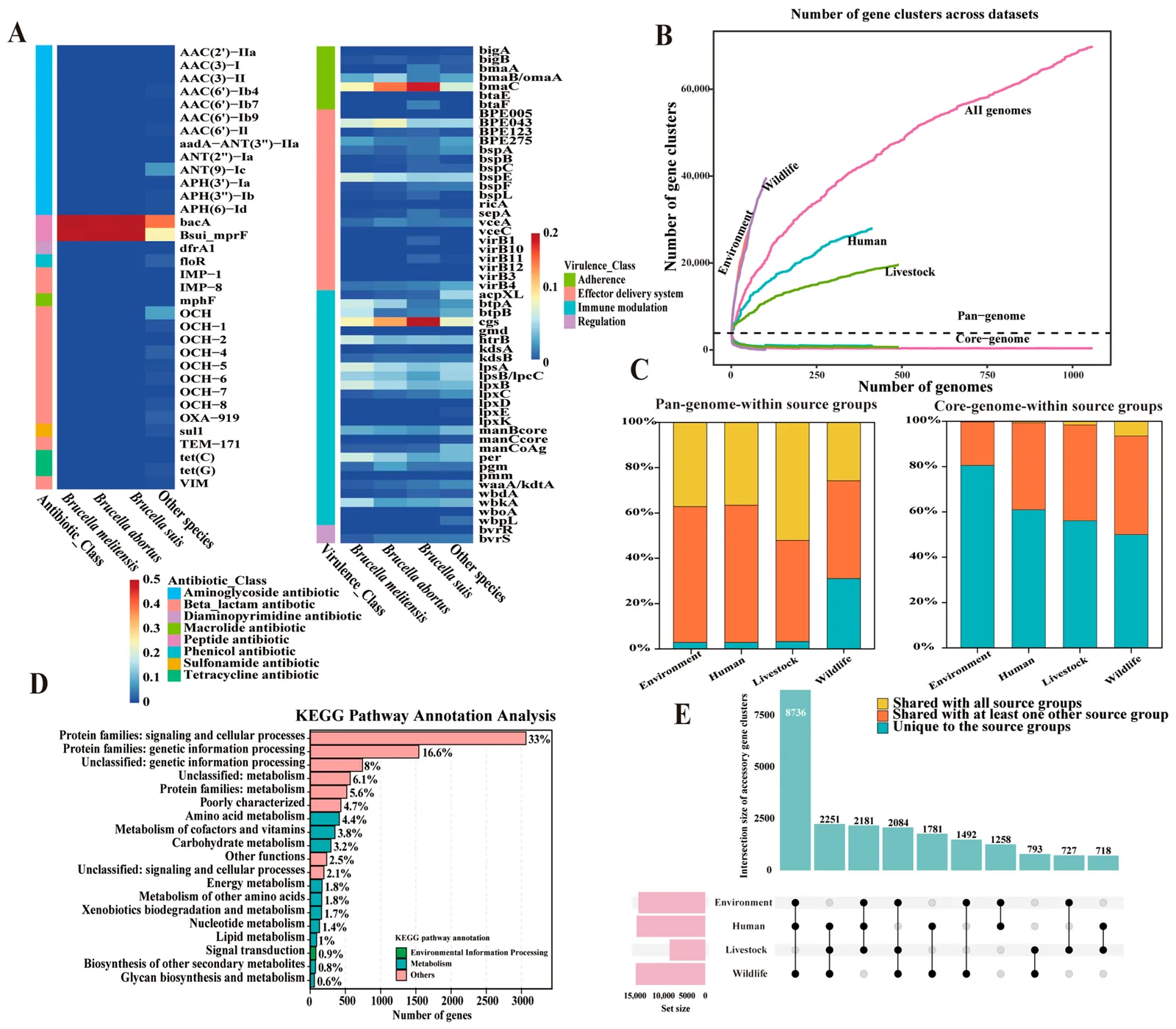
Species-specific ARG and VF distributions and pan-genome characteristics of *Brucella* spp. genomes. (**A**) Prevalence profiles of selected ARGs and VFs among *B. melitensis*, *B. abortus*, and *B. suis*. Color gradients indicate the proportion of genomes carrying each gene. (**B**) Pan-genome and core-genome accumulation curves showing changes in gene cluster numbers with increasing genome number. (**C**) Proportions of shared and source-group-specific gene clusters in the pan-genome and core-genome sets defined within different isolation-source groups. (**D**) KEGG pathway annotation of gene clusters, showing the major functional categories represented in the dataset. (**E**) UpSet analysis showing shared and source-specific accessory gene clusters among environmental, human, livestock, and wildlife isolates. In panel E, black dots indicate the source groups included in each intersection, whereas light-gray dots indicate groups not included; vertical connecting lines denote intersections involving multiple source groups.

**Figure 5:**
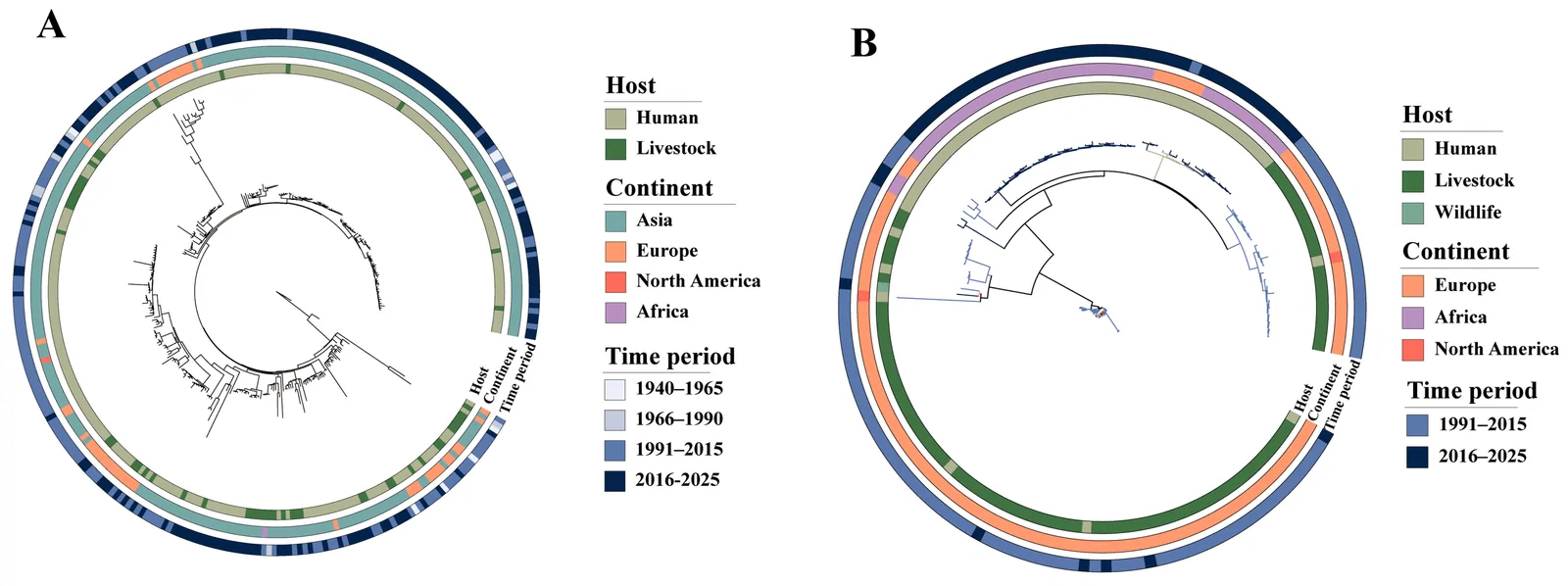
Maximum-likelihood phylogenetic trees of ST8 and ST11 isolates based on core-genome alignment. (**A**) Phylogenetic tree of ST8 isolates. (**B**) Phylogenetic tree of ST11 isolates. The circular annotation rings represent, from the inner to outer layers, host origin, continent, and isolation period.

**Figure 6:**
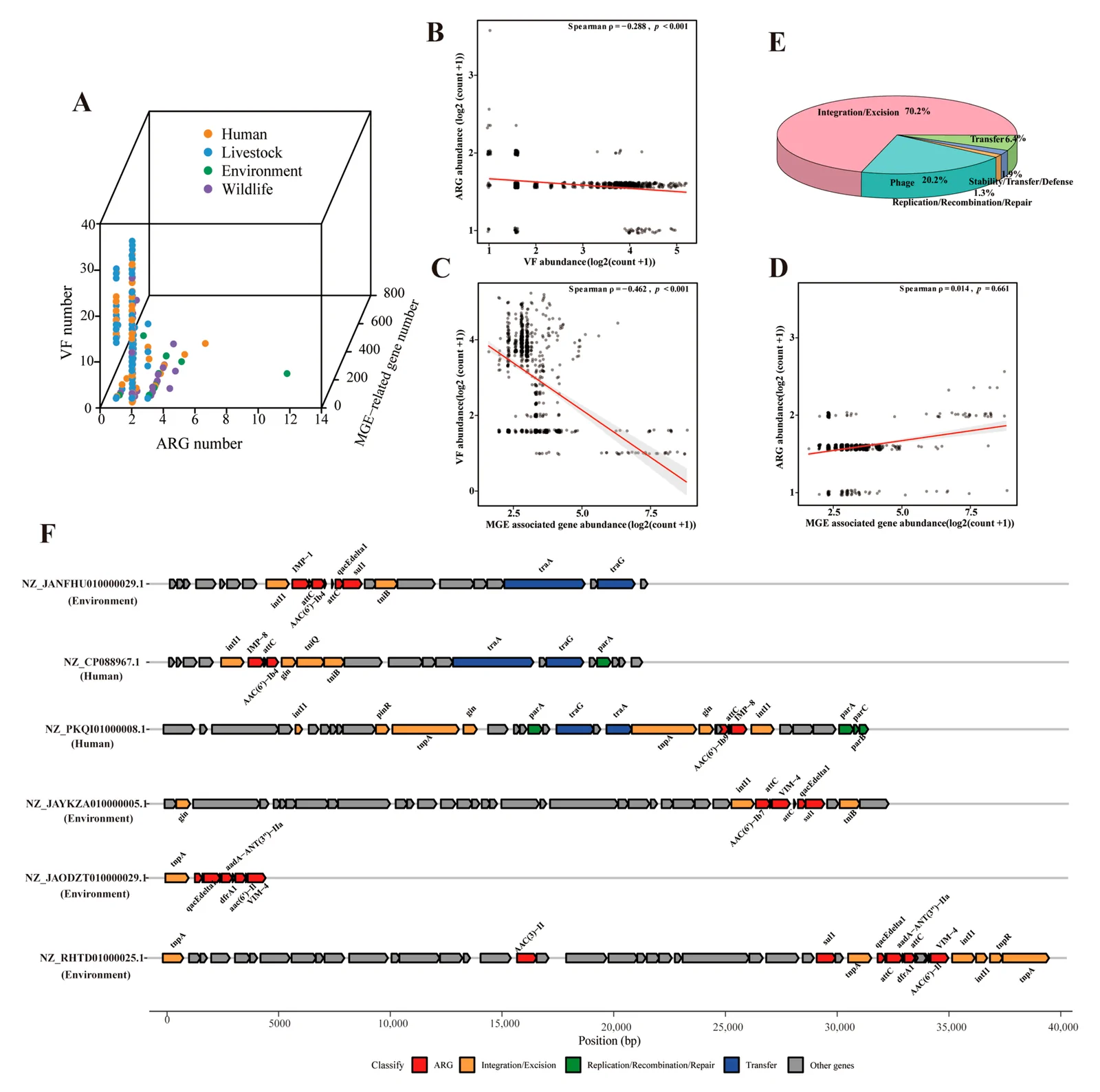
Comprehensive analysis of ARGs, VFs, MGE-associated genes, and related genomic features in *Brucella* spp. genomes. (**A**) Three-dimensional distribution of ARG abundance, VF abundance, and MGE-associated gene abundance across *Brucella* spp. genomes from different sources. (**B**–**D**) Spearman correlation analyses between ARG abundance and VF abundance (**B**), ARG abundance and MGE-associated gene abundance (**C**), and VF abundance and MGE-associated gene abundance (**D**). (**E**) Functional composition of MGE-associated genes. (**F**) Genomic organization of the aminoglycoside and β-lactam resistance genes and associated genes in representative contigs, showing gene order and orientation.

## Data Availability

The genome assemblies analyzed in this study were obtained from publicly available databases. The accession numbers and associated metadata for the genomes included in this study are provided in Table S1, while information on excluded genomes and the corresponding reasons for exclusion is provided in Table S11. Additional processed data and analysis results supporting the findings of this study are provided in Tables S2–S10 and S12 and Figures S1–S15 of the Supplementary Materials.

## Supplementary Materials

The following supporting information can be downloaded at: https://www.mdpi.com/article/10.3390/microorganisms14102132/s1, Supplementary Methods; Figure S1: Metadata composition of the *Brucella* spp. genomic dataset; Figure S2: Compositional profiles of ARGs and VFs across different isolate metadata groups; Figure S3: Shannon diversity of ARG and VF profiles in *Brucella* spp. genomes; Figure S4: Functional classification of ARGs and VFs in *Brucella* spp. genomes; Figure S5: Enrichment patterns of ARGs and VFs across isolate metadata groups; Figure S6: Presence/absence profiles of ARGs and VFs across *Brucella* species; Figure S7: Pangenome accumulation patterns across temporal and continental subsets of the global *Brucella* genome dataset; Figure S8: Temporal signal assessment of *Brucella* genomes using TempEst; Figure S9: Abundance and diversity of MGE-associated gene annotations across isolate metadata groups; Figure S10: Sensitivity analyses of ARG, VF, and MGE-associated gene correlations across major species and ST groups; Figure S11: Co-localization network and genomic environments of ARGs and MGE-related genes; Figure S12: Genome-quality sensitivity analysis of ARG profiles; Figure S13: Genome-quality sensitivity analysis of VF profiles; Figure S14: Genome-quality sensitivity analyses of pan-genome and phylogenetic patterns; Figure S15: Genome-quality sensitivity analysis of MGE-associated profiles; Table S1: Metadata of the 1059 *Brucella* spp. genomes included in this study; Table S2: Abundance matrix of ARGs in 1059 *Brucella* spp. genomes; Table S3: Abundance matrix of VFs in 1059 *Brucella* spp. genomes; Table S4: PERMANOVA and multivariate dispersion analyses of ARG and VF profiles across host, geographic, and temporal groups; Table S5: Pan-genome characteristics and Heaps’ law parameters of *Brucella* spp. genomes across different host and geographic groups; Table S6: Pairwise SNP distance matrix for ST8 *Brucella* spp. genomes; Table S7: Pairwise SNP distance matrix for ST11 *Brucella* spp. genomes; Table S8: Sensitivity analyses of ARG, VF, and MGE-associated gene correlations across major species and ST groups; Table S9: Genomic context annotations of ARGs and MGE-associated genes used for genomic environment visualization; Table S10: Characteristics of core-gene alignments and phylogenetic informative sites for ST8 and ST11 lineages; Table S11: Summary of excluded genomes and reasons for exclusion during dataset selection; Table S12: Summary of genome-quality sensitivity analyses comparing the original 1059-genome dataset and the high-quality (HQ) subset (*n* = 800).

## Abbreviations

The following abbreviations are used in this manuscript:

AMR	Antimicrobial resistance
ARGs	Antimicrobial resistance genes
ARB	Antibiotic-resistant bacteria
FDR	False discovery rate
HGT	Horizontal gene transfer
IMG	Integrated Microbial Genomes
MGEs	Mobile genetic elements
ML	Maximum likelihood
MLST	Multilocus sequence typing
NCBI	National Center for Biotechnology Information
PERMANOVA	Permutational multivariate analysis of variance
STs	Sequence types
VFs	Virulence factors
