# Supplementary material for: Global Genomic Analysis of Brucella spp. Reveals Antimicrobial Resistance, Virulence, and Mobile Genetic Element Profiles from a One Health Perspective
Source: Microorganisms. 2026 Sep 22;14(10):2132. doi: 10.3390/microorganisms14102132 (PMC13707526; doi:10.3390/microorganisms14102132)
Supplement: Supplementary file 1 [file microorganisms-14-02132-s001.zip › supplementary-figures.pdf]

Supplementary File Information

Global Genomic Analysis of Brucella spp. Reveals Antimicrobial Resistance, Virulence, and Mobile Genetic Element Profiles from a One Health Perspective

Chenyang Han [1], Tao He [1], Yafei Liang [1], Bo Yang [1], Wenjuan Gong [1], Haihong Hao [2], Jinliang Sheng [1,*] and Yu Pan [1,*]

[1] Xinjiang Production and Construction Corps Key Laboratory for Efficient Utilization of Non-Grain Feed Resources, International Joint Research Center for Healthy Animal Farming, College of Animal Science and Technology, Shihezi University, Shihezi 832003, China; 18238863132@163.com (C.H.); ht960704@163.com (T.H.); 13899390597@163.com (Y.L.); 18089337984@163.com (B.Y.); 19399877826@163.com (W.G.)
[2] National Reference Laboratory of Veterinary Drug Residues, Huazhong Agricultural University, Wuhan 430070, China; haohaihong@mail.hzau.edu.cn
* Correspondence: sjlshz@126.com (J.S.); yp117@shzu.edu.cn (Y.P.)

This file contains Supplementary Methods, Tables S1–S12, and Figures S1–S15.

Supplementary Methods

Integration of the SARG and CARD reference databases

CARD was used as the primary ARG reference source, whereas SARG was used as a supplementary source. During database integration, SARG entries were cross-referenced against CARD using ARO identifiers. If an ARO was already represented in CARD, the corresponding CARD entry was retained, and the overlapping SARG entry was excluded to avoid duplicate representation. SARG entries corresponding to AROs not represented in CARD were retained as supplementary reference entries. The resulting nonredundant combined ARG reference database was subsequently used for BLASTP annotation. For each query sequence, only the top-ranked target hit was retained using -max_target_seqs 1; therefore, each query sequence contributed at most one retained ARG annotation. Distinct query sequences within the same genome were evaluated independently, including potentially paralogous ARG-like sequences. ARG abundance was calculated as the number of retained ARG annotations per genome.

Selection of the Roary identity threshold

During preliminary parameter evaluation, Roary was initially run using a 90% protein identity threshold. Under this setting, the analysis did not yield a usable core-gene alignment for the complete multi-species dataset. A more permissive 80% identity threshold was therefore evaluated. This setting produced the complete output required for downstream analysis and was used in the final pan-genome analysis. The final Roary analysis was performed using the parameters -i 80 -e --mafft.

Species and ST level sensitivity analyses

To evaluate whether the observed correlations among ARG abundance, VF abundance, and MGE-associated gene abundance were affected by population structure, additional sensitivity analyses were performed across major species and ST groups. Spearman rank correlation tests were conducted separately within each group.

Only groups with at least 30 genomes were included for stratified analyses. Correlations were not calculated when insufficient within-group variation was present in either variable. P values from the stratified analyses were adjusted using the Benjamini–Hochberg false discovery rate (FDR) method.

Core-gene clustering and alignment handling

Paralogous genes and potentially duplicated gene sequences were handled within the standard Roary clustering workflow. No additional manual exclusion of duplicated genes or ambiguously aligned regions was performed.

Assessment of temporal signal and time-scaled phylogenetic inference

To evaluate whether the dominant ST lineages were suitable for time-scaled Bayesian phylogenetic inference, temporal signal was assessed using TempEst based on root-to-tip regression between genetic distance and sampling year. The analyses were performed separately for ST8 and ST11 using the corresponding maximum likelihood trees. Because both lineages showed weak temporal structure, Bayesian time-scaled phylogenetic reconstruction was not included in the final analysis, and evolutionary relationships were interpreted primarily based on the maximum likelihood phylogeny. Exploratory Bayesian phylogenetic analyses were initially attempted for the dominant ST lineages; however, due to weak temporal signal and limited support for reliable molecular dating, the resulting time-scaled estimates were not retained for interpretation.

Genome-quality sensitivity analysis

To assess the potential influence of genome assembly quality on the principal analyses, an additional sensitivity analysis was performed using a high-quality (HQ) subset of the original 1,059-genome dataset. Genomes with completeness ≥95% and contamination ≤5% were retained, resulting in an HQ subset of 800 genomes. ARG, VF, MGE-associated gene, pan-genome, and phylogenetic analyses were repeated using the same definitions, annotation procedures, and analytical parameters as those applied to the primary dataset.

For ARG and VF sensitivity analyses, differences in mean annotation abundance between the HQ subset and the original dataset were evaluated using permutation tests

based on 5,000 random subsets of 800 genomes drawn without replacement from the original 1,059-genome dataset. Nested bootstrap resampling with 5,000 iterations was used to estimate 95% confidence intervals for differences in mean annotation abundance. Concordance of functional composition was evaluated using Bray–Curtis similarity, while gene-prevalence profiles were compared using Bray–Curtis similarity, Pearson correlation, and absolute prevalence differences. Distinct feature richness was additionally compared with its distribution obtained from 5,000 random 800-genome subsets of the original dataset. For MGE-associated features, the same nested-subset framework was used for abundance and prevalence comparisons, and P values for multiple functional-category comparisons were adjusted using the Benjamini–Hochberg FDR method.

Pan-genome analysis was repeated for the HQ subset using the same Roary workflow and clustering parameters as in the primary analysis, and pan-genome accumulation patterns, gene-cluster distributions, and fitted accumulation parameters were compared between datasets. For phylogenetic sensitivity analysis, the ST8 maximum-likelihood phylogeny was reconstructed using the 277 ST8 genomes retained in the HQ subset, following the same workflow as in the primary analysis; the original ST8 analysis included 299 genomes. For ST11, all 139 genomes included in the original analysis met the HQ criteria; therefore, quality filtering did not alter the ST11 analysis set, and a separate reconstructed ST11 tree was not required. For ST8, phylogenetic concordance between the original and HQ analyses was further evaluated using the 277 genomes shared between the two datasets. Topological concordance was assessed using the Robinson–Foulds distance, and concordance of pairwise patristic distances was evaluated using Pearson and Spearman correlations. The ST8 sensitivity analysis was used to assess whether the major lineage structure and broad host- and geographic-associated clustering patterns were retained after quality filtering. No additional molecular-clock analysis was performed because the previously observed temporal signal was weak.

Supplementary Results

Of the 1,059 genomes in the primary dataset, 800 met the HQ criteria of completeness ≥95% and contamination ≤5%. ARG profiles changed only slightly after quality filtering: mean ARG annotation abundance was 2.07 per genome in the original dataset and 2.01 in the HQ subset. The dominant ARG-class composition and major ARG prevalence patterns were retained, with peptide antibiotic resistance-associated annotations remaining predominant and *bacA* and *Bsui_mprF* remaining widely distributed (Figure S12).

VF profiles were also broadly similar between datasets. Mean VF annotation abundance increased from 9.00 to 9.72 per genome, but the major functional composition and prevalence patterns remained comparable, including immune modulation, effector delivery system, and adherence-associated categories (Figure S13).

Reanalysis of the pan-genome using the 800 HQ genomes retained the increasing accumulation pattern and the interpretation of an open pan-genome. For ST8, the HQ phylogeny retained the broad lineage structure and host- and geographic-associated clustering patterns observed in the original analysis (Figure S14). All 139 ST11 genomes included in the original analysis met the HQ criteria, so the ST11 analysis set remained unchanged after quality filtering.

MGE-associated profiles were more sensitive to genome-quality filtering. The distribution of unique MGE-related annotations per genome differed between the original and HQ datasets (permutation $P < 0.001$). However, integration/excision- and phage-associated functions remained highly prevalent in both datasets, whereas replication/recombination/repair, stability/transfer/defense, and transfer-associated categories showed lower prevalence in the HQ subset (Figure S15). Overall, quality filtering affected several quantitative genomic features, including rare ARGs, accessory/pan-genome features, and particularly MGE-associated annotations, while the dominant ARG and VF profiles, open pan-genome interpretation, and broad phylogenetic structure were retained.

Supplementary Tables

Table S1. Metadata of the 1,059 *Brucella* spp. genomes included in this study.

Table S2. Abundance matrix of ARGs in 1,059 *Brucella* spp. genomes.

Table S3. Abundance matrix of VFs in 1,059 *Brucella* spp. genomes.

Table S4. PERMANOVA and multivariate dispersion analyses of ARG and VF profiles across host, geographic, and temporal groups.

Table S5. Pan-genome characteristics and Heaps' law parameters of *Brucella* spp. genomes across different host and geographic groups.

Table S6. Pairwise SNP distance matrix for ST8 *Brucella* spp. genomes.

Table S7. Pairwise SNP distance matrix for ST11 *Brucella* spp. genomes.

Table S8. Sensitivity analyses of ARG, VF, and MGE-associated gene correlations across major species and ST groups.

Table S9. Genomic context annotations of ARGs and MGE-associated genes used for genomic environment visualization.

Table S10. Characteristics of core-gene alignments and phylogenetic informative sites for ST8 and ST11 lineages.

Table S11. Summary of excluded genomes and reasons for exclusion during dataset selection.

Table S12. Summary of genome-quality sensitivity analyses comparing the original 1,059-genome dataset and the high-quality (HQ) subset (n = 800).

Supplementary Figures

Figure S1. Metadata composition of the *Brucella* spp. genomic dataset**.**

Figure S2. Compositional profiles of ARGs and VFs across different isolate metadata groups.

Figure S3. Shannon diversity of ARG and VF profiles in *Brucella* spp. genomes.

Figure S4. Functional classification of ARGs and VFs in *Brucella* spp. genomes.

Figure S5. Enrichment patterns of ARGs and VFs across isolate metadata groups.

Figure S6. Presence/absence profiles of ARGs and VFs across *Brucella* species.

Figure S7. Pangenome accumulation patterns across temporal and continental subsets of the global *Brucella* genome dataset.

Figure S8. Temporal signal assessment of *Brucella* genomes using TempEst.

Figure S9. Abundance and diversity of MGE-associated gene annotations across isolate metadata groups.

Figure S10. Sensitivity analyses of ARG, VF, and MGE-associated gene correlations across major species and ST groups.

Figure S11. Co-localization network and genomic environments of ARGs and MGE-related genes.

Figure S12. Genome-quality sensitivity analysis of ARG profiles.

Figure S13. Genome-quality sensitivity analysis of VF profiles.

Figure S14. Genome-quality sensitivity analyses of pan-genome and phylogenetic patterns.

Figure S15. Genome-quality sensitivity analysis of MGE-associated profiles.

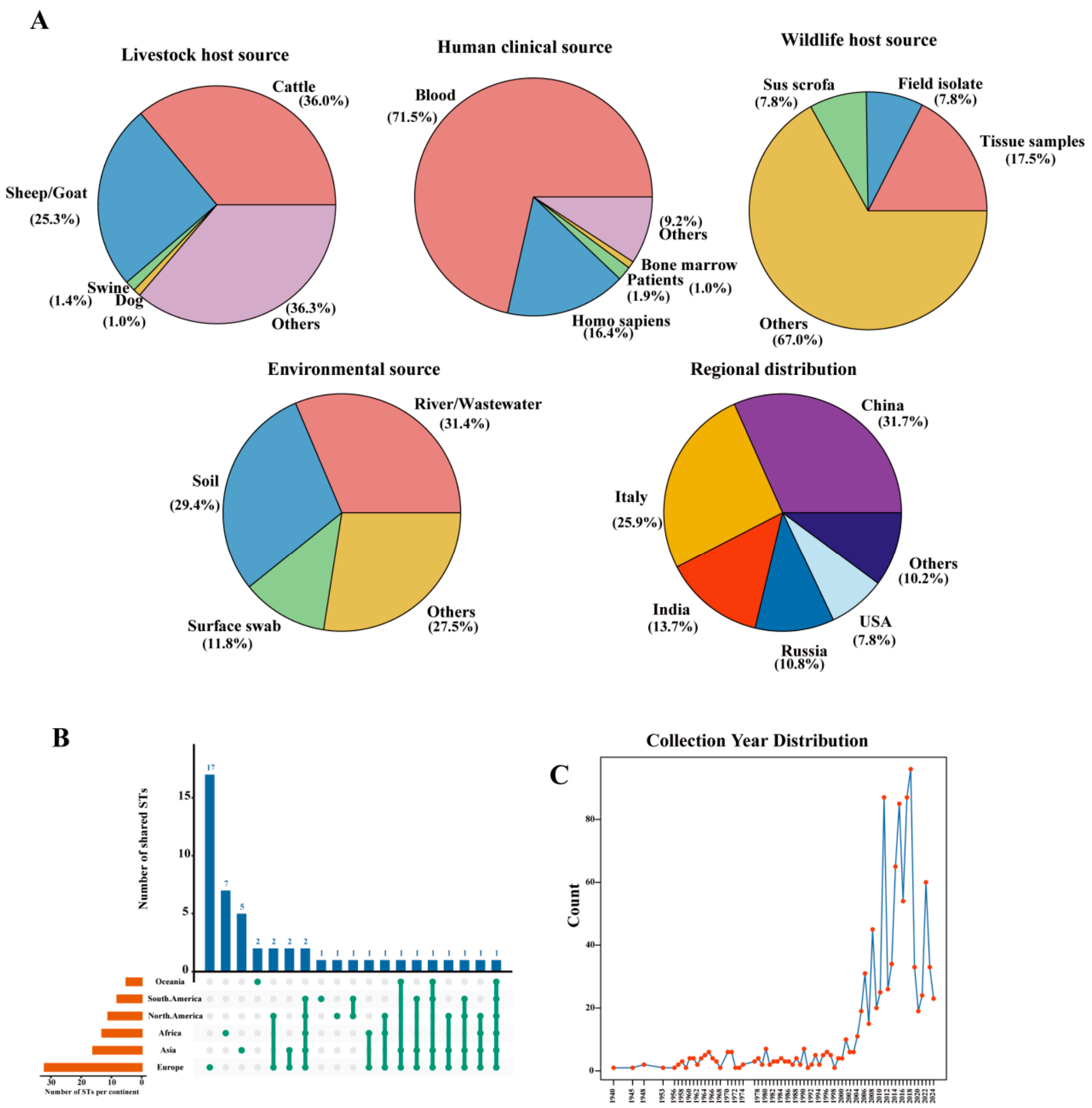

Figure S1. Metadata composition of the *Brucella* spp. genomic dataset. (A) Pie charts showing the distribution of isolates according to livestock host source, human clinical source, wildlife host source, environmental source, and major country/region of origin. Percentages indicate the proportion of each category within the corresponding group. **(B)** UpSet plot showing the overlap of STs across continents. The upper bars represent the number of STs shared by specific continental combinations, while the left bars represent the total number of STs detected in each continent. Green connected dots indicate the continents included in each ST intersection. **(C)** Collection year distribution of the analyzed *Brucella* spp. genomes.

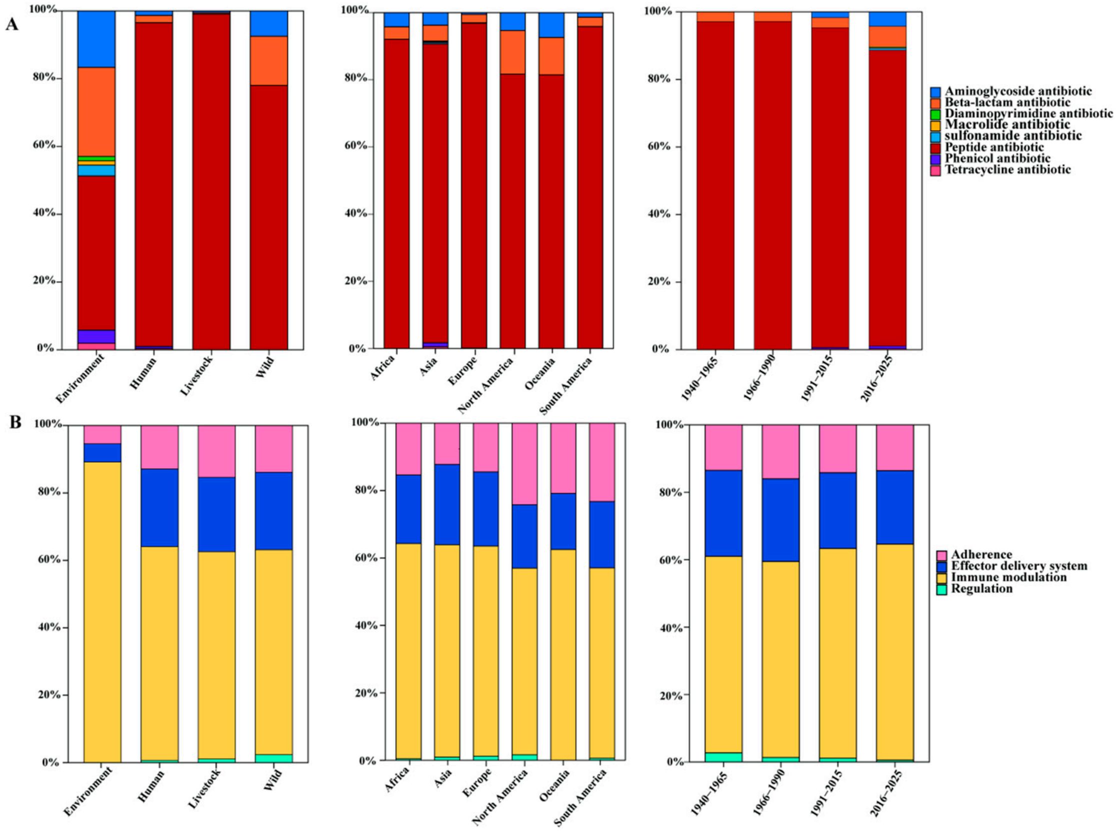

Figure S2. Compositional profiles of ARGs and VFs across different isolate metadata groups. (A) Proportional distribution of ARG classes by source, region, and collection period. **(B)** Proportional distribution of virulence factor categories in the same groups. For each metadata group, percentages were calculated as the number of ARG or VF annotation entries assigned to each category divided by the total number of ARG or VF annotation entries within that group, respectively.

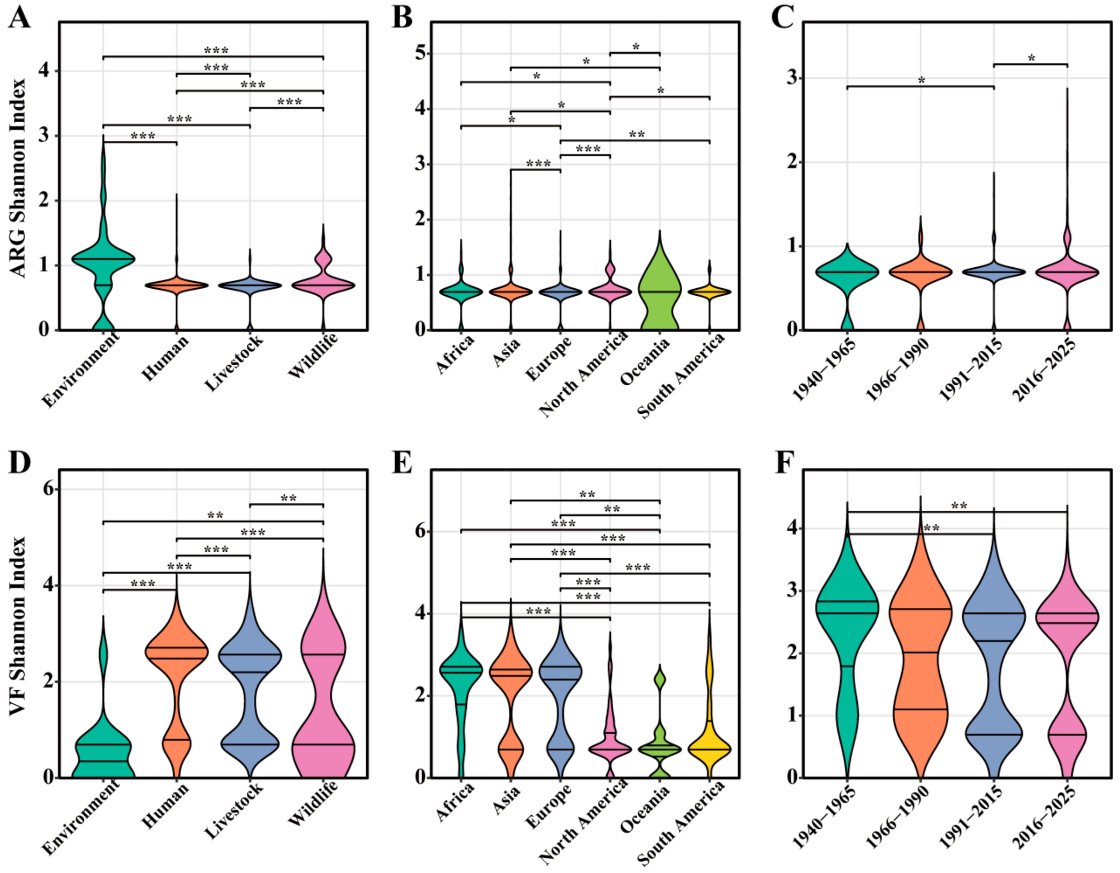

Figure S3. Shannon diversity of ARG and VF profiles in *Brucella* spp. genomes. ARG Shannon index was compared across source **(A)**, continent **(B)**, and isolation period **(C)**, while the VF Shannon index was compared across source **(D)**, continent **(E)**, and isolation period **(F)**. Statistical significance is indicated as *$P < 0.05$, **$P < 0.01$, and ***$P < 0.001$.

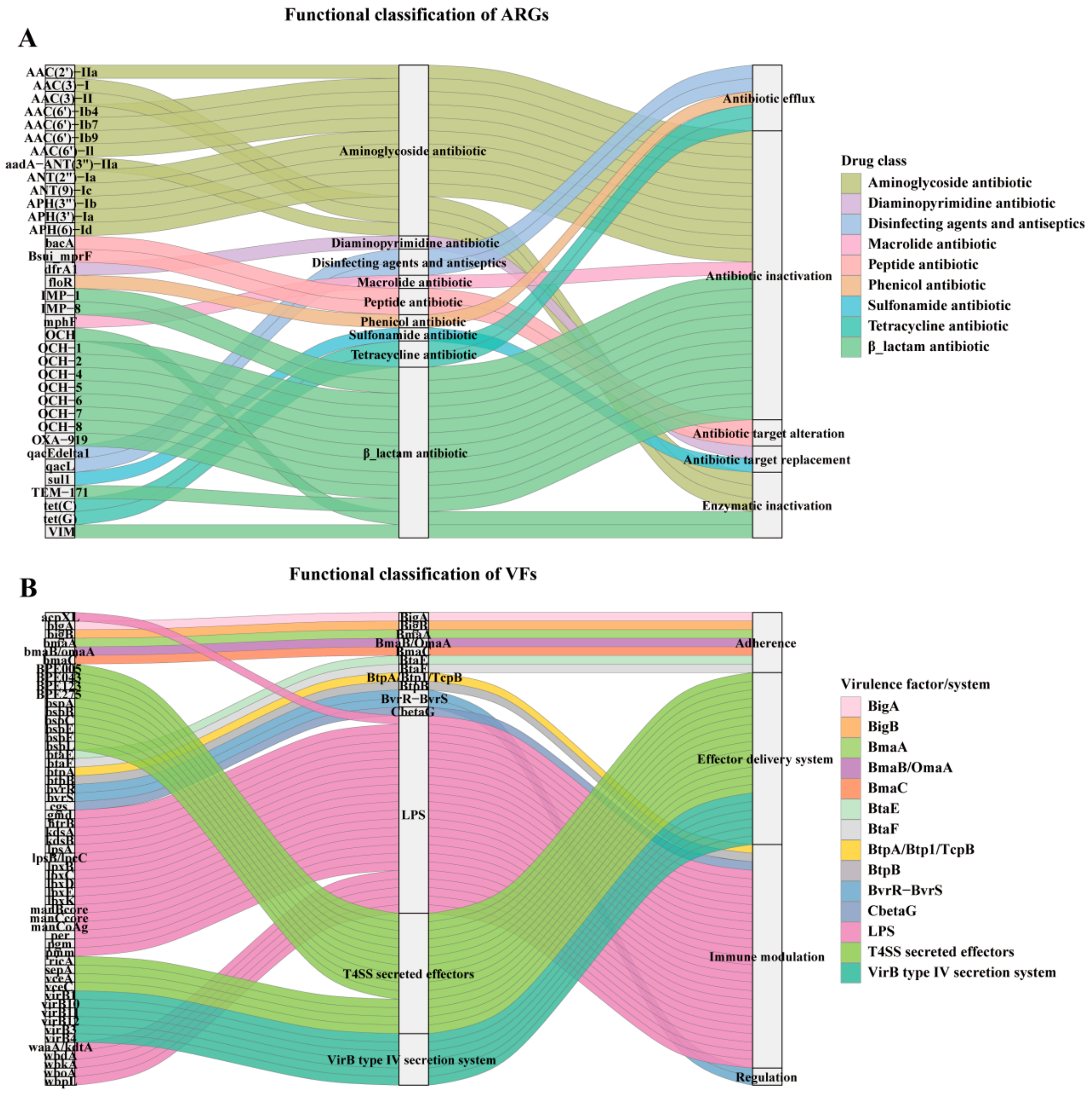

Figure S4. Functional classification of ARGs and VFs in *Brucella* spp. genomes. **(A)** Sankey diagram showing the classification of ARGs according to antibiotic class and resistance mechanism. The left nodes represent individual ARGs, the middle nodes indicate antibiotic classes, and the right nodes indicate resistance mechanisms. **(B)** Sankey diagram showing the classification of VFs according to virulence factor/system and functional category. The left nodes represent individual VF genes, the middle nodes indicate virulence factor systems, and the right nodes indicate functional classes. Flow widths represent the relative frequencies of pooled ARG or VF annotation entries across all analyzed genomes.

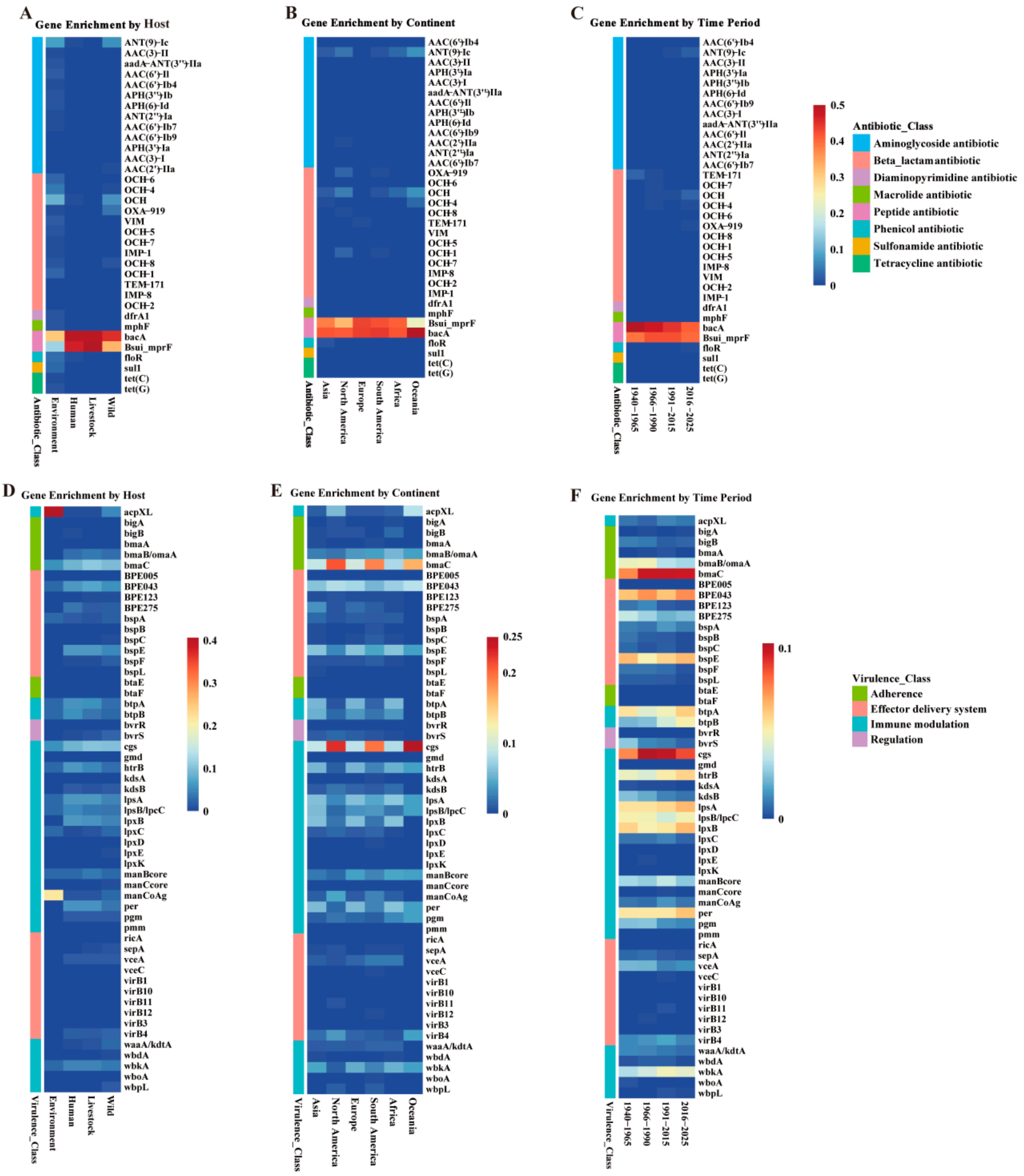

Figure S5. Enrichment patterns of ARGs and VFs across isolate metadata groups. (A-C) Heatmaps showing the enrichment of ARGs across host/source groups, continents, and collection periods. **(D-F)** Heatmaps showing the enrichment of virulence factor genes across the same metadata groups. Color intensity represents the enrichment level, and side annotation bars indicate antibiotic or virulence functional classes.

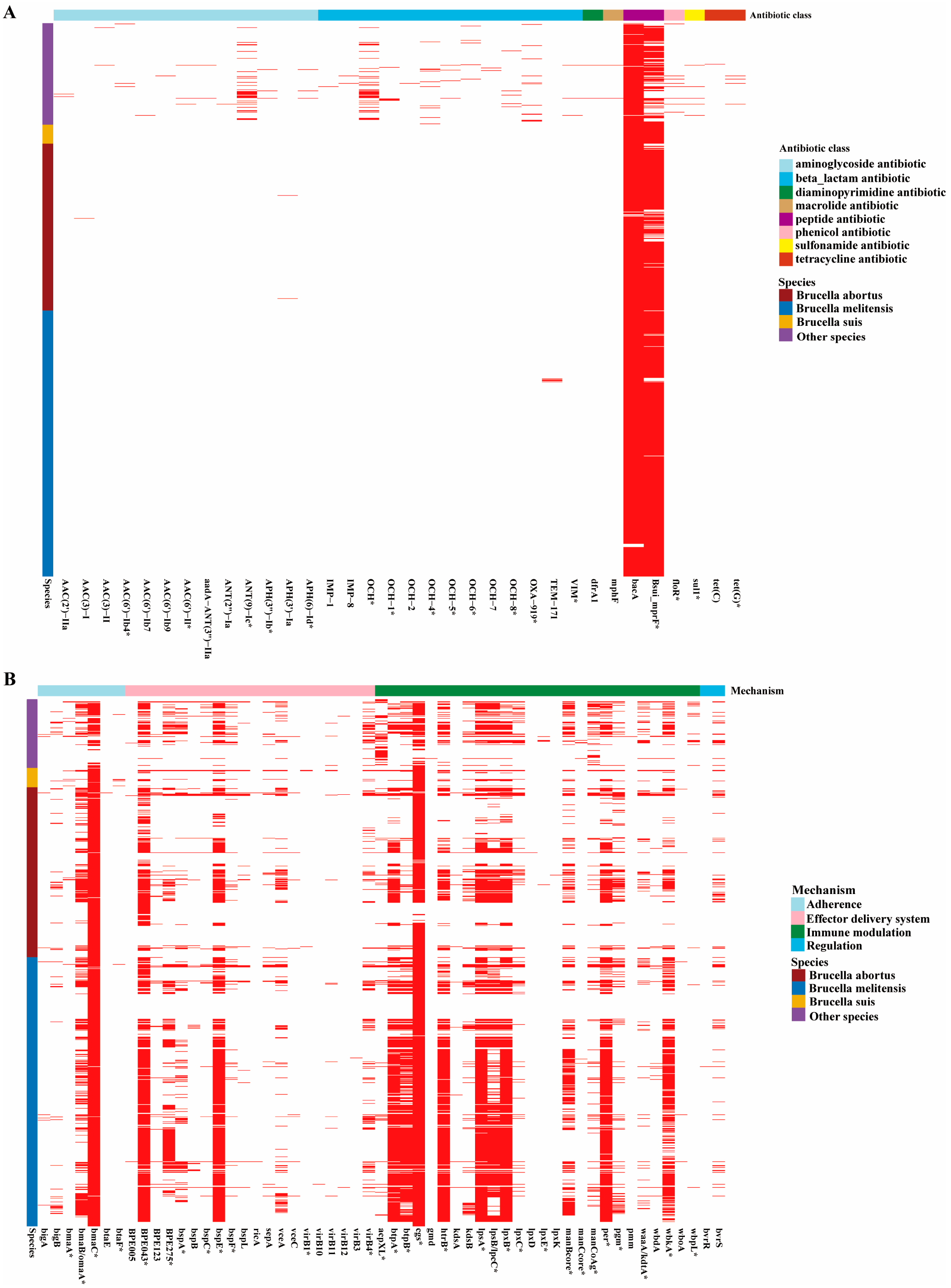

Figure S6. Presence/absence profiles of ARGs and VFs across *Brucella* species. (A) Distribution of antimicrobial resistance genes (ARGs) categorized by antibiotic class. **(B)** Distribution of virulence factor genes (VFs) categorized by functional mechanism. Isolates are grouped by species, including *B. abortus*, *B. melitensis*, *B. suis*, and other *Brucella* species. Red indicates gene presence and white indicates absence. Differential gene distribution among species was assessed using Fisher's exact test, with FDR-adjusted $P < 0.05$ marked by asterisks.

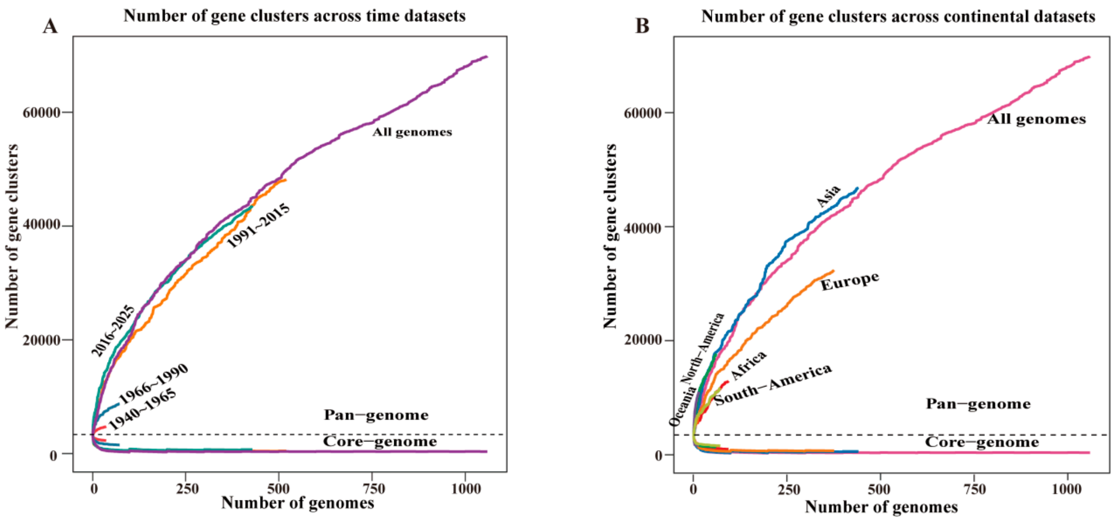

Figure S7. Pangenome accumulation patterns across temporal and continental subsets of the global *Brucella* genome dataset. (A) Gene cluster accumulation curves across collection-period subsets. **(B)** Gene cluster accumulation curves across continental subsets. The curve based on all genomes is shown for comparison, with pan-genome and core-genome trends indicated.

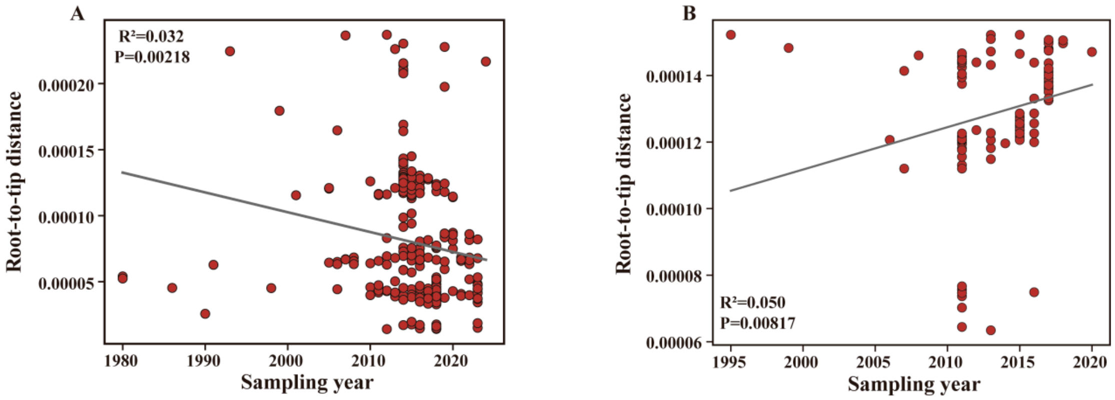

Figure S8. Temporal signal assessment of *Brucella* genomes using TempEst. Root-to-tip genetic distances were regressed against sampling years to evaluate the temporal structure of different sequence types. **(A)** Temporal signal analysis of ST8 genomes. **(B)** Temporal signal analysis of ST11 genomes. The coefficient of determination (R^2) and regression P value are shown in each panel.

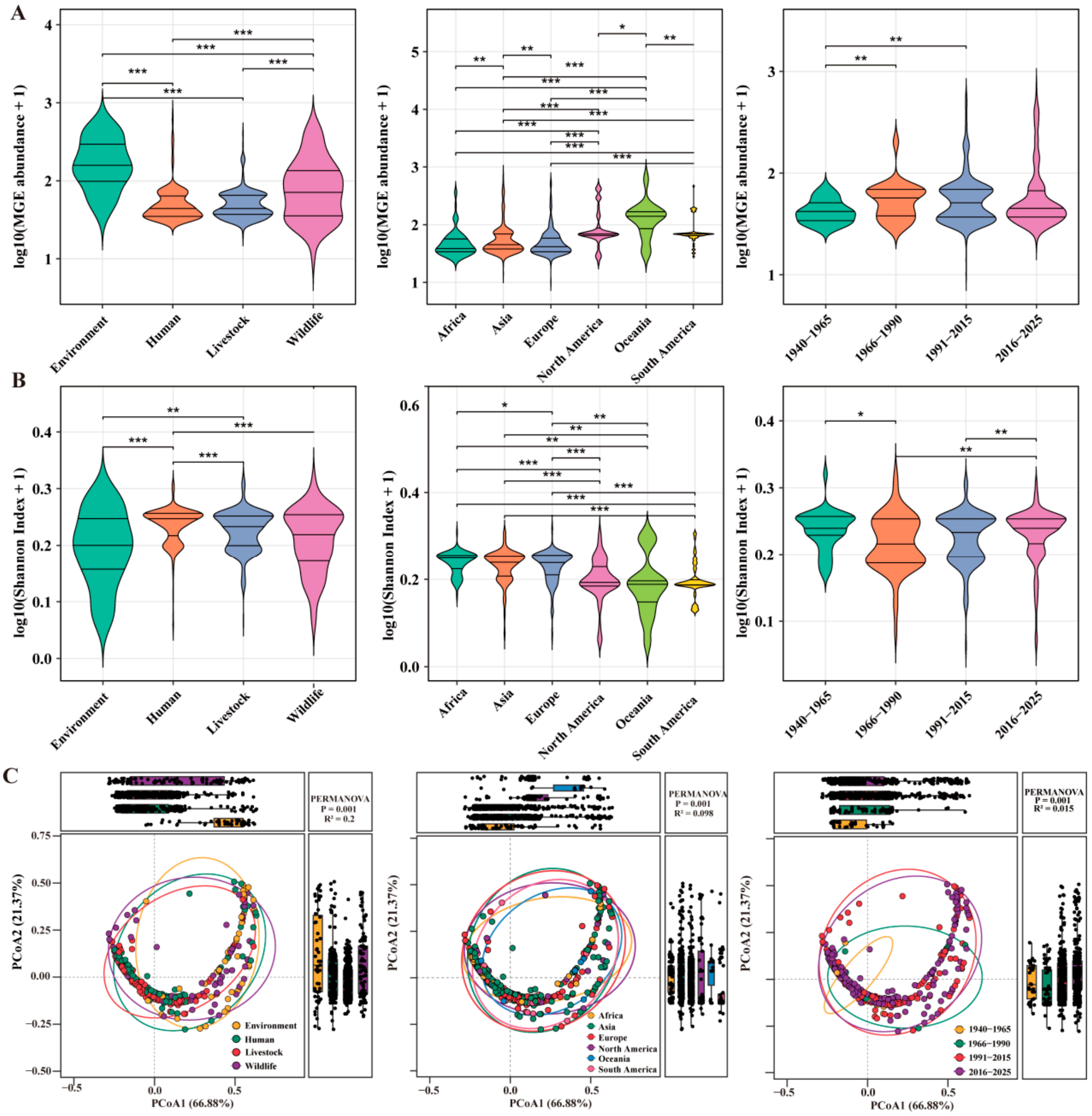

Figure S9. Abundance and diversity of MGE-associated gene annotations across isolate metadata groups. (A) MGE-associated gene annotation counts per genome, shown as log10-transformed values, across source types, continents, and collection periods. **(B)** Shannon diversity index of MGE-associated gene profiles across the same groups. **(C)** PCoA showing differences in MGE-associated gene composition by source type, continent, and collection period. PERMANOVA P values and R^2 values are shown in each panel.

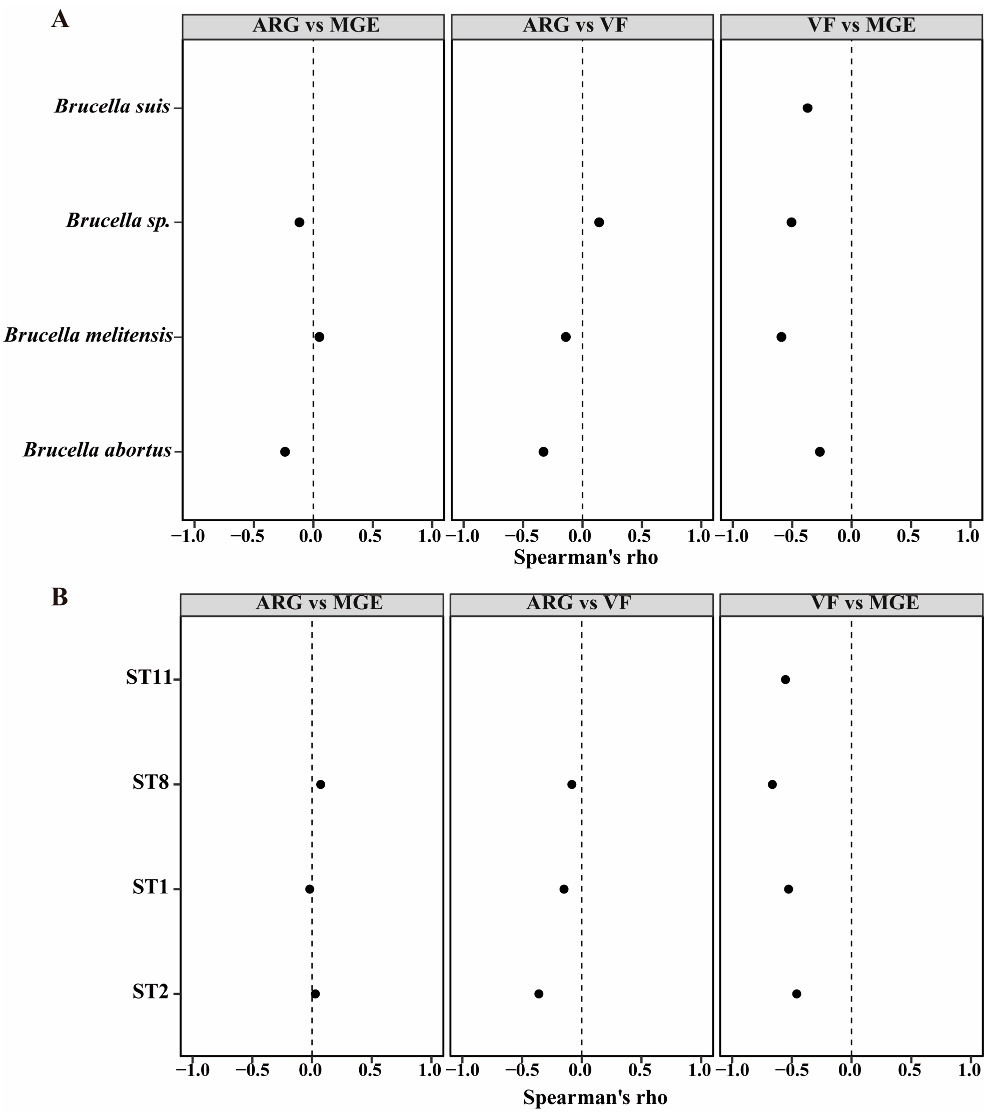

Figure S10. Sensitivity analyses of ARG, VF, and MGE-associated gene correlations across major species and ST groups. (A) Correlation patterns within major *Brucella* species groups. **(B)** Correlation patterns within major ST groups. Spearman correlation coefficients (rho) are shown for each comparison. Only groups with sufficient sample size and variation were included.

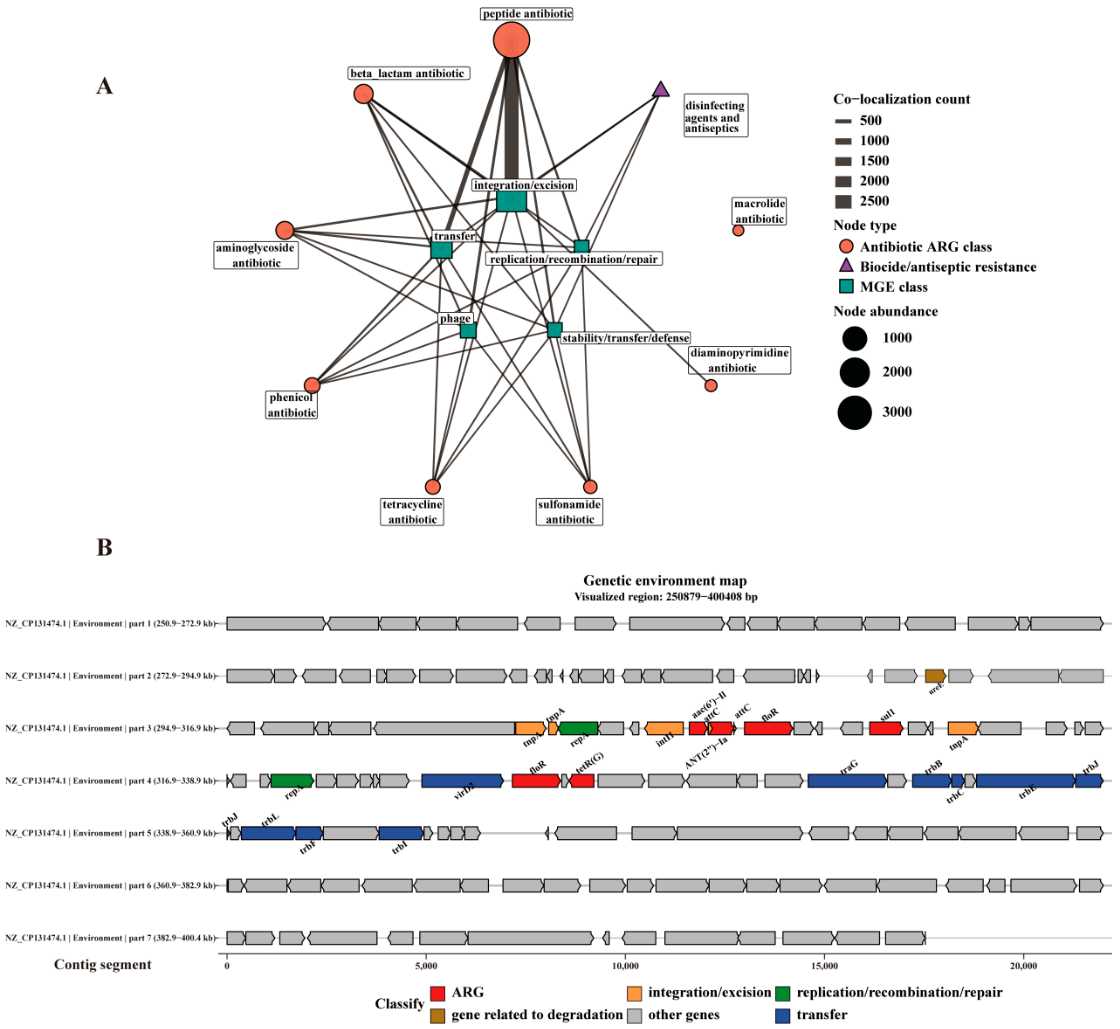

Figure S11. Co-localization network and genomic environments of ARGs and MGE-related genes. (A) Network showing co-localization relationships among ARG classes, biocide/antiseptic resistance, and MGE functional classes. Node size represents gene abundance, and edge width indicates co-localization count. **(B)** Genetic environment map of representative ARG-containing contig regions. Arrows indicate predicted genes and their orientations, with colors denoting ARGs, MGE-related functional categories, and other genes.

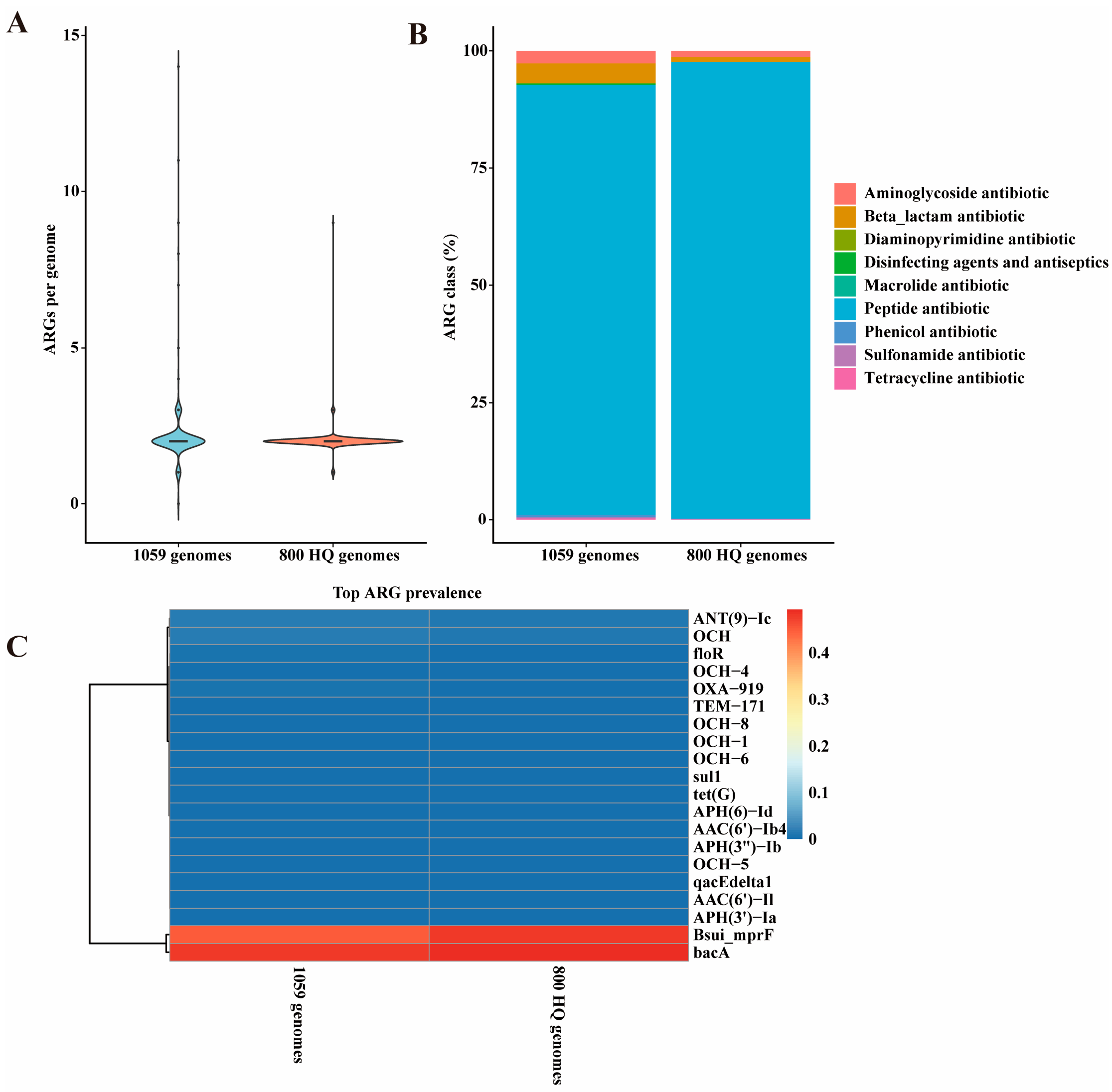

Figure S12. Genome-quality sensitivity analysis of ARG profiles. (A) Distribution of ARG annotations per genome in the original dataset (n = 1,059) and the high-quality (HQ) subset (n = 800). **(B)** Relative composition of ARG classes in the original and HQ datasets. **(C)** Heatmap showing the prevalence of the major ARGs in the two datasets. Color intensity represents gene prevalence.

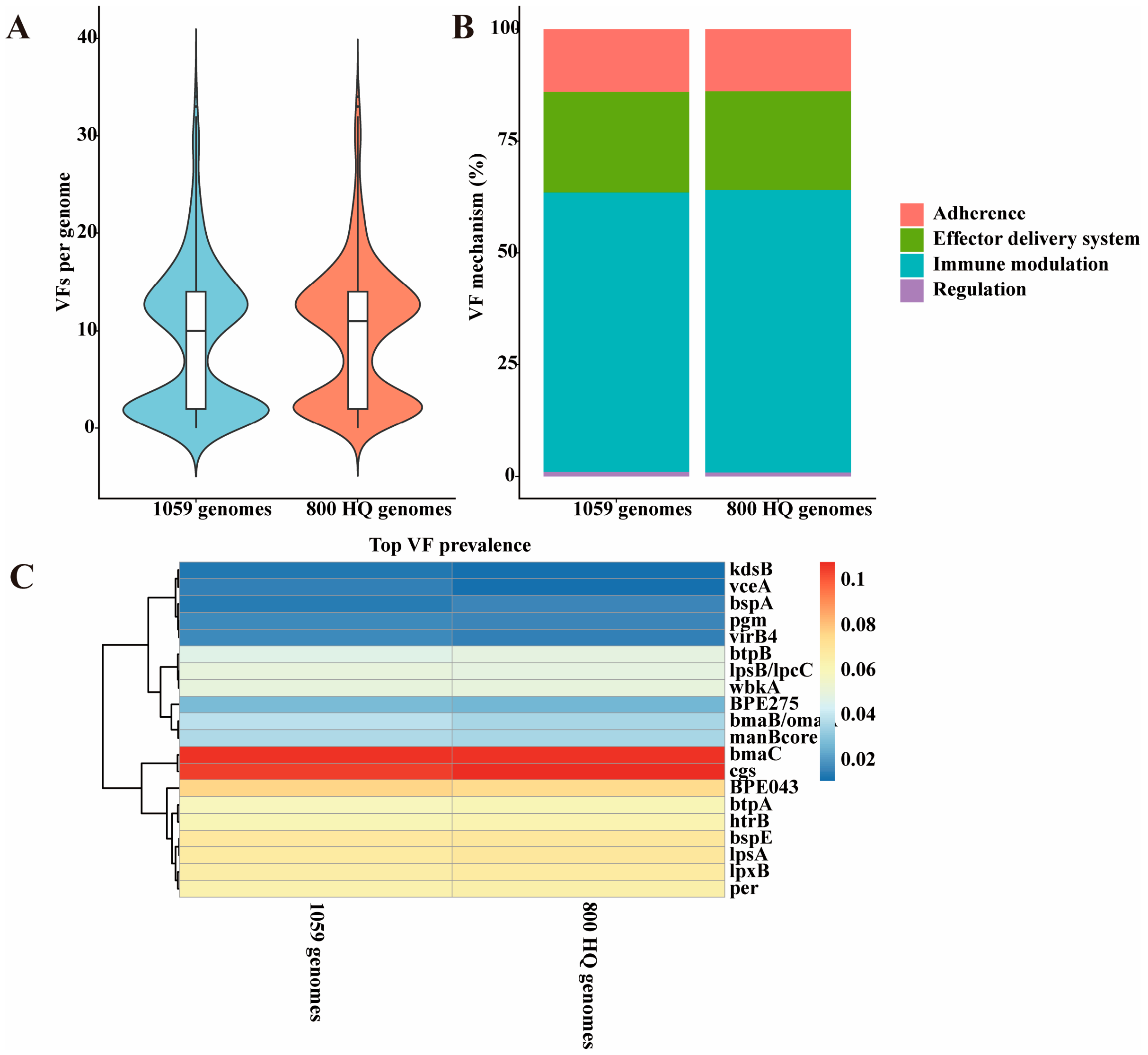

Figure S13. Genome-quality sensitivity analysis of VF profiles. (A) Distribution of VF annotations per genome in the original dataset (n = 1,059) and the high-quality (HQ) subset (n = 800). **(B)** Relative composition of VF functional mechanisms in the two datasets. **(C)** Heatmap showing the prevalence of the major VFs in the original and HQ datasets. Color intensity represents gene prevalence.

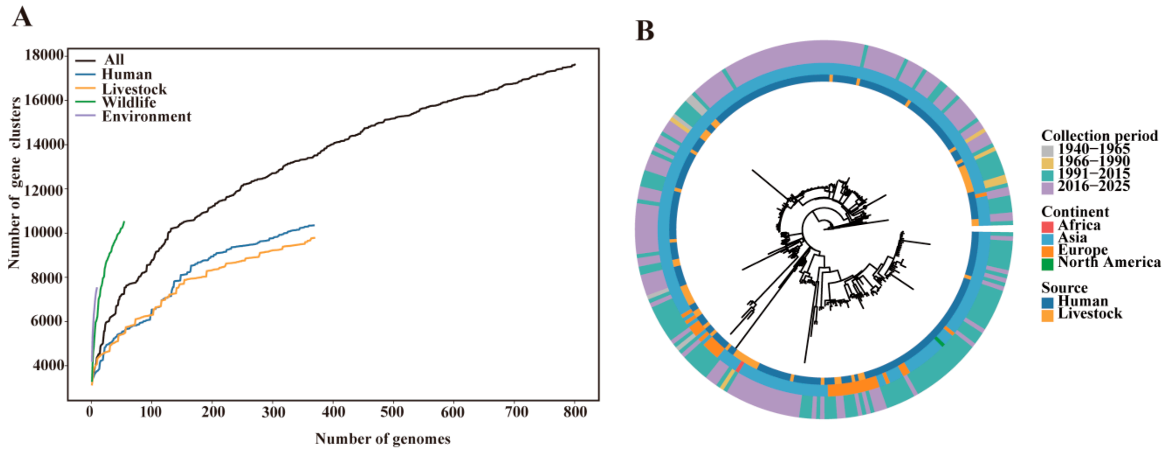

Figure S14. Genome-quality sensitivity analyses of pan-genome and phylogenetic patterns. (A) Pan-genome accumulation curves for the HQ genome subset and genomes stratified by isolation source, including human, livestock, wildlife, and environmental isolates. The number of gene clusters is plotted against the number of genomes included. **(B)** Maximum-likelihood phylogenetic tree reconstructed using the 277 ST8 genomes retained in the HQ subset. Circular annotation rings indicate collection period, continent, and isolation source.

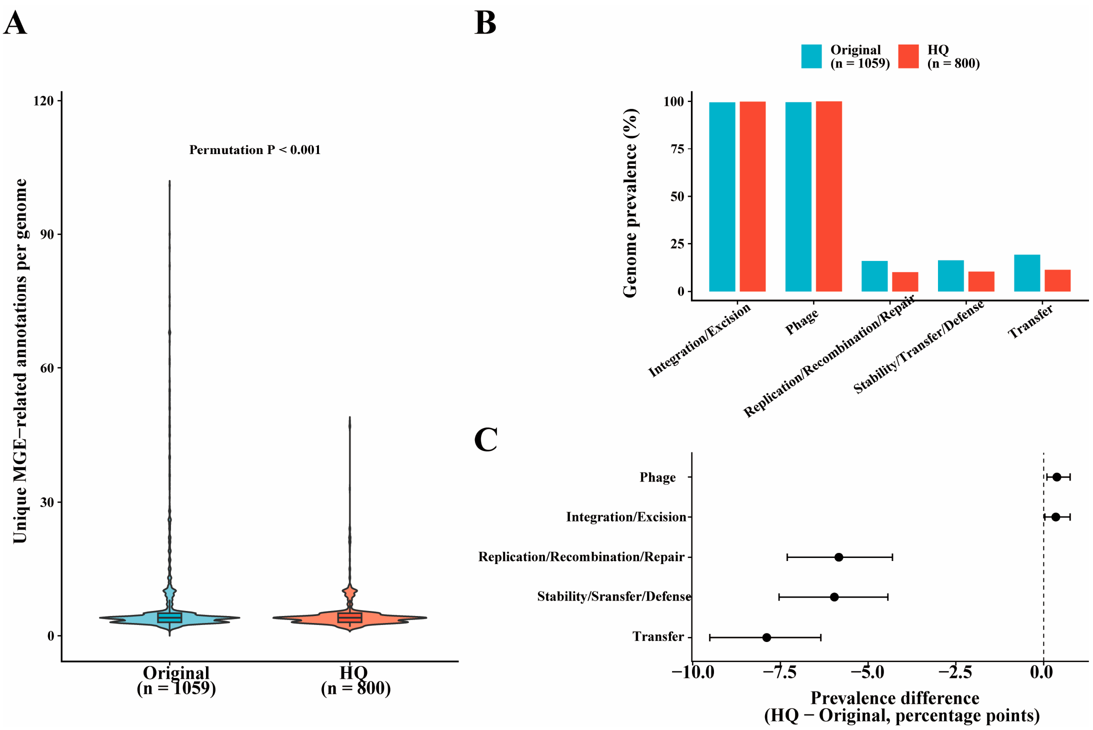

Figure S15. Genome-quality sensitivity analysis of MGE-associated profiles. (A) Distribution of unique MGE-related annotations per genome in the original dataset (n = 1,059) and the HQ subset (n = 800). The difference was evaluated using a permutation test. **(B)** Genome-level prevalence of the major MGE functional categories in the original and HQ datasets. **(C)** Differences in functional-category prevalence between the HQ subset and the original dataset (HQ − Original), with error bars indicating 95% confidence intervals; the dashed vertical line indicates no difference.
